# KM-DBSCAN: an enhanced density and centroid based border detection framework for data reduction towards green AI

**DOI:** 10.1038/s41598-026-40062-z

**Published:** 2026-03-27

**Authors:** Mohamed Yasser AboElsaad, Mohamed Farouk, Hatem A. Khater

**Affiliations:** 1 Department of Computer Science, College of Computing and Information Technology, Arab Academy of Science Technology and Maritime Transport, Alexandria, Egypt; 2 Computer and Systems Engineering Department, Faculty of Engineering, Horus University Egypt, New Damietta, Egypt

**Keywords:** Green AI, Red AI, Data reduction, K-means, DBSCAN, SVM, MLP, CNN, Computational biology and bioinformatics, Engineering, Mathematics and computing

## Abstract

Green AI aims to design and train machine learning models while taking into consideration sustainable resource usage without sacrificing model efficiency. The exponential growth of training data has led to results in increasing computational cost and energy consumption. Techniques like pruning, quantization, and knowledge distillation are used to shrink AI models. Data reduction is one of these techniques that enhances both the training speed up factor and the green AI score. To overcome these challenges, we introduce KM-DBSCAN, a new data clustering algorithm for intelligent data reduction. It aims to combine the geometric simplicity of K-Means with the density-awareness and noise resilience of DBSCAN to enhance the performance and the efficiency of data clustering for better border detection even in overlapping scenarios. The effect of data reduction has been examined on training and testing different machine learning models including SVM, MLP and CNN on six benchmark datasets which are Banana, USPS, Adult9a, Collision, Dry Bean and Melanoma. KM-DBSCAN achieved up to 90% data reduction, training speedups up to 3.6$\times$ to 6900$\times$, and carbon emission 0.0219 g to 5.374 g , while preserving competitive accuracy (e.g., 90.39% accuracy in melanoma classification using only 28.7% of the training data, with just 0.0061% accuracy loss and a 71.65% reduction in carbon emissions compared to training on the full dataset). These results demonstrate that KM-DBSCAN enables efficient and environmentally-conscious learning without compromising predictive performance.

## Introduction

In recent years, scientists and researchers have increasingly believed that utilizing large-scale datasets enhances the accuracy of traditional machine learning algorithms and deep learning models regardless of the associated costs. While this belief holds some truth, it often overlooks the significant computational complexity and memory requirements of such algorithms. As the size of the data increases, so too does the computational burden, leading to longer training times and greater demand for hardware components such as storage, RAM, and GPUs. Consequently, energy consumption rises, resulting in higher carbon emissions that negatively impact the environment. This phenomenon is commonly referred to in recent literature as Red Artificial Intelligence (Red AI)^1^. In response to these growing concerns, the concept of green artificial intelligence (Green AI) has evolved as a counterpoint to Red AI, emphasizing developing AI systems with high performance measures and environmentally sustainable. Introduced by Schwartz et al.^1^, Green AI promotes a broader evaluation framework that considers energy efficiency, hardware utilization, and carbon footprint alongside traditional metrics like accuracy. As deep learning models continue to scale in size and complexity, their training and deployment increasingly contribute to substantial environmental costs. Green AI encourages researchers to adopt strategies that reduce computational demands without sacrificing model performance. These strategies include key techniques such as data reduction, architectural optimization, and transfer learning, each contributing to reducing the environmental footprint of machine learning. Data reduction focuses on minimizing the training dataset by eliminating redundant or less informative instances, thereby reducing both memory usage and computational time. Architectural optimization aims to design more efficient neural network structures that require fewer parameters and operations, leading to lower energy consumption. Transfer learning leverages knowledge from pre-trained models, significantly cutting down training requirements on new tasks. Among these, data reduction plays a particularly crucial role in our study, as it offers a model-agnostic approach to improving efficiency. Specifically, it encompasses techniques such as dimensionality reduction and numerosity reduction^2^, which allow for substantial savings in computational resources while preserving the core information necessary for effective generalization thus aligning closely with the goals of Green AI.

**Fig. 1:**
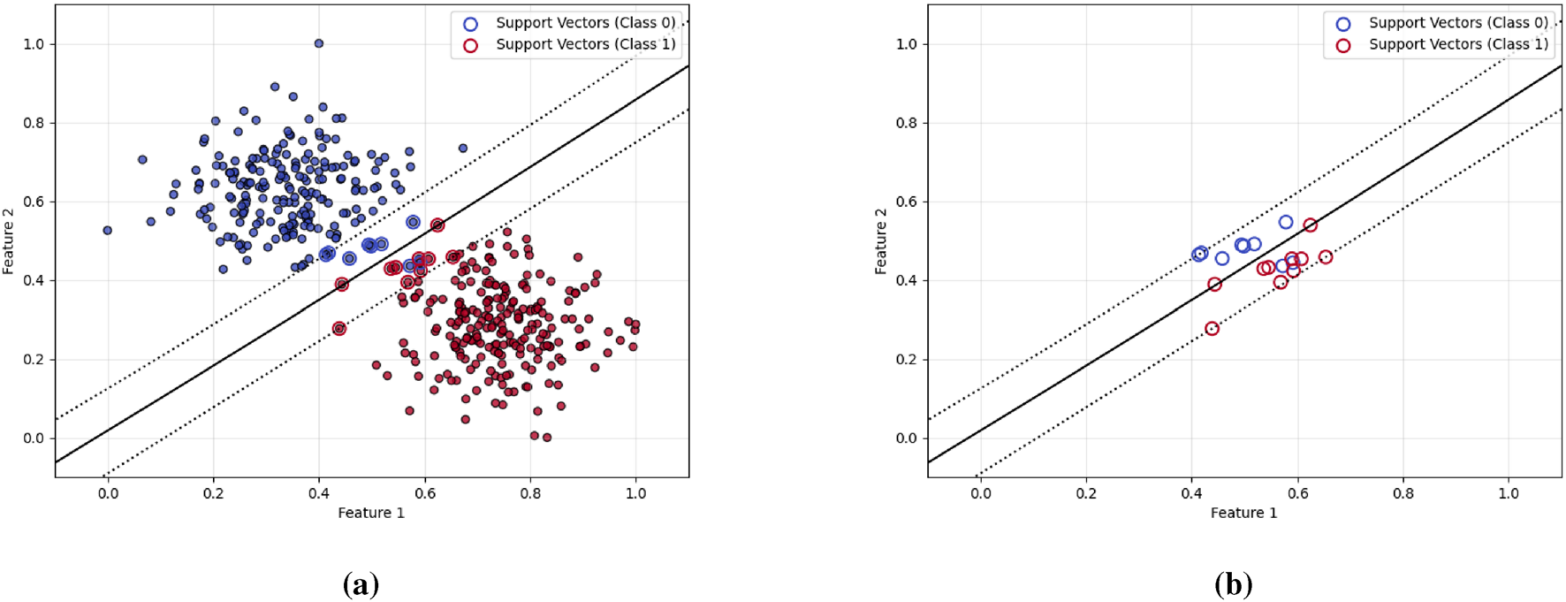
Comparison of SVM decision boundaries: (**a**) SVM trained on the complete dataset versus (**b**) SVM trained only on the support vectors extracted from sub-figure (**a**) as a reduced set.

Dimensionality reduction is a preprocessing step in machine learning, that is used to reduce the number of original features while keeping the substantial structure of the data^3^. Two widely adopted methods for dimensionality reduction are principal component analysis (PCA) and uniform manifold approximation and projection (UMAP). PCA is a linear transformation technique that reorients the original data into a new coordinate system, such that the direction of highest variance becomes the first principal component, the second highest variance aligns with the second component, and so on. Mathematically, PCA computes the eigenvectors of the covariance matrix and projecting the data onto the top-k components to get a reduced representation^4^. If the data has nonlinear structures, PCA can not effectively extract the underlaying hidden structure of the data. kernel PCA is an alternative where some research is focused on reducing the nonlinearity within the the data as a linear approximation for the problem^5^. In contrast, UMAP is a nonlinear, manifold-based method aims to preserve both local and global data structures. It builds a high-dimensional fuzzy topological structures for the data and then tries to optimize a low-dimensional embedding using stochastic gradient descent. UMAP achieves this by minimizing the cross-entropy between the high-dimensional and low-dimensional fuzzy simplicial sets, resulting in an effective low-dimensional projection. While PCA is computationally efficient and more interpretable due to its linear nature, UMAP often provides superior performance in capturing complex, nonlinear relationships within high-dimensional datasets^6^. Both techniques are widely used for tasks such as data visualization, noise reduction, and preprocessing steps before classification or clustering. On the other hand, numerosity reduction techniques such as instance selection or sampling focus on reducing the number of data points themselves, rather than the number of features. These methods aim to eliminate redundant or noisy instances while preserving a representative and informative subset of the original dataset^7^, as shown in Fig. 1, the left subfigure (a) presents the SVM training on an entire dataset, including both informative and potentially redundant instances. In contrast, subfigure (b) demonstrates SVM training only on a subset of the data as the support vectors extracted from sub-figure (a), which form a compact, informative subset of the data. Notably, the decision boundaries in both cases are nearly identical, indicating that removing redundant samples does not significantly affect the classification performance. This highlights the potential of instance selection techniques in choosing a subset of the data that is sufficient for constructing the decision boundary to reduce data size and computational cost without compromising model quality.

To address the aforementioned challenge, we propose KM-DBSCAN, a novel hybrid clustering algorithm that integrates K-Means with DBSCAN. Initially designed as an unsupervised clustering technique, KM-DBSCAN aims to enhance the time complexity and cluster detection by combining the centroid initialization strengths of K-Means with the density-based robustness of DBSCAN. This integration enables more precise identification of dense regions and separation of distinct data clusters, improving the overall clustering performance. Beyond its clustering capability, KM-DBSCAN also serves as an effective data reduction strategy, selecting representative border data points from clusters as a density based approach to form a compact yet informative subset of the original dataset. This reduced subset is then used for training downstream models, contributing to improved computational efficiency while preserving and in some cases improving classification performance. The effectiveness of this approach has been validated across a various of machine learning models, including traditional classifiers such as Support Vector Machines (SVM) and Multi-Layer Perceptrons (MLP), as well as deep learning models like Convolutional Neural Networks (CNNs).

In the medical domain, we particularly emphasize the application of our method in the early diagnosis of melanoma an aggressive form of skin cancer through the analysis of dermoscopic images. Automated classification using dermoscopic images has become an important tool to assist dermatologists in clinical decision-making. However, this task faces several challenges, one of the most significant being the substantial computational resources required for training deep learning models on large-scale medical data. Inspired by the work of Waheed et al.^8^, who proposed a CNN-based system that emulates the diagnostic reasoning of dermatologists by learning discriminative lesion features, we incorporate KM-DBSCAN as a data preprocessing step to eliminate redundant training instances. This integration aims to streamline the training pipeline and reduce computational overhead while maintaining accuracy, making it more feasible to deploy such diagnostic tools in real-world clinical environments, especially where computational resources are limited.

The rest of this paper is structured as follows: Section 2 outlines the background of the employed techniques along with a review of related work. Section 3 describes the proposed method, while Sect. 4 explains the experimental setup, evaluation procedure and results. Section 5 discusses the main findings. Section 6 concludes the study. Finally, Sect. 7 outlines its limitations and directions for future research.

## Background and related work

Unsupervised clustering is a fundamental task in machine learning, aiming to group unlabeled data into coherent groups based on similarity or data distribution. Two prominent algorithms in this domain are K-Means and DBSCAN, each with distinct mathematical foundations and performance characteristics. K-Means clustering seeks to partition data into *k* clusters by minimizing the intra-cluster variance. Given a dataset $X = \{x_1, x_2, \ldots , x_n\}$ and a predefined number of clusters *k*, the algorithm iteratively updates the cluster centroids $\{\mu _1, \mu _2, \ldots , \mu _k\}$ by minimizing the objective function:

1 $$\begin{aligned} \min \sum _{i=1}^{k} \sum _{x_j \in C_i} \Vert x_j - \mu _i\Vert ^2 \end{aligned}$$

where $C_i$ denotes the set of points assigned to cluster *i*, and $\Vert \cdot \Vert$ is the Euclidean norm. While efficient and scalable, K-Means assumes convex, spherical clusters of similar density and fails to handle noise or overlapping clusters effectively. It is also sensitive to initialization choice of the number of clusters *k*. DBSCAN is a density-based algorithm that discovers clusters by identifying dense regions of points in the data. A point *p* is a *core point* if it has at least *MinPts* points within a neighborhood radius $\varepsilon$:

2 $$\begin{aligned} \left| \left\{ x_i \in X \;\vert \; \Vert x_i - p\Vert \le \varepsilon \right\} \right| \ge \text {MinPts} \end{aligned}$$

Points reachable from core points but not themselves dense are classified as *border points*, while those that do not satisfy either condition are labeled as *noise* or outliers. Unlike K-Means, DBSCAN can discover clusters of arbitrary shape and is robust to noise. However, it struggles in datasets with clusters of varying density and overlapping boundaries, where tuning $\varepsilon$ and *MinPts* becomes non-trivial. In overlapping regions, DBSCAN may incorrectly merge nearby clusters or mislabel border points as noise. Clustering has a wide range of applications, including image segmentation and anomaly detection, with data reduction being one of the most significant. Several studies have explored data reduction techniques to improve the efficiency of machine learning models. Shen et al.^9^ proposed a two-stage data reduction method tailored for SVM training by analyzing data distributions within clusters. The core assumption of their approach is that data points near cluster centroids typically do not include support vectors (SVs) and can therefore be removed, whereas sparser points located at the cluster periphery are more likely to be SVs and should be retained. In the first stage, clusters are generated using algorithms such as K-Means, and irrelevant clusters are filtered out using class labels and the Max-Min Cluster Distance (MMCD) method, which measures each cluster’s proximity to an estimated hyperplane. In the second stage, the remaining data points are sorted based on their distance to the cluster centroid, and the Fisher Discriminant Ratio (FDR) is employed to identify and eliminate non-essential points. This two-stage reduction strategy significantly improves SVM training speed and memory usage while maintaining classification performance. To enhance computational efficiency, a faster variant called FIFDR is also introduced, which accelerate this process, which involves using different iterative step lengths to narrow the range of distance densities calculated through the FDR method. Birzhandi and Youn^10^ introduced the Clustering-Based Convex Hull (CBCH) algorithm, in which k-means is used to group the training data into k partition based on the structure and size of the dataset. The efficiency of the algorithm is affected by the number of clusters and the centroids initialization. The resulting clusters are categorized as either singular or nonsingular. Singular is containing data points from a single class and nonsingular is containing points from multiple classes. Nonsingular clusters are used in full to construct the reduced set as they most probably contain the support vectors. In contrast, singular clusters are reduced using the Quickhull algorithm, which constructs convex hulls to identify informative boundary points, while discarding the remaining interior points. The final reduced dataset, consisting of these vertices and all data from nonsingular clusters, is used for SVM training. Aslani and Seipel^11^ proposed an algorithm for Border Point extraction based on Locality-Sensitive Hashing (BPLSH). Multiple hash buckets are utilized to group the data points using families of locality-sensitive hashing functions. The number of shared buckets between each pair of data points is used to generate similarity index. Border points are retained and identified as points that have close neighbors belonging to the opposite class, while only one representative point is preserved in homogeneous regions. The performance of BPLSH depends heavily on the granularity of the hashing partitions. Specifically, data points are considered neighbors if they share at least one bucket, and are considered very close if they share all buckets across all hash families. Liu et al.^12^ proposed the Shell Extraction (SE) algorithm. This method iteratively eliminates inner-class data points based on geometric proximity to class centroids. Initially, the centroid of each class is computed, and a reduction sphere is defined around it using a certain radius. A user defined parameter is used to scale the average distance from the centroid to the class points to find the radius value. Data points within this radius are removed. New centroids are recalculated from the remaining data points, and the reduction spheres expand. The process continues until the number of retained samples drops below a predefined threshold. The method assumes spherical class distributions, and its performance is highly sensitive to the choice of radius and expansion parameters. Ghaffari et al.^13^ introduced a multi-stage data reduction method focused on minimizing boundary complexity. In the first stage, harmful samples whose labels conflict with the majority of their neighbors are removed using a threshold-guided search tree. In the second stage, outliers outside any neighborhood are discarded. Subsequently, boundary points are selected as mutually closest samples from different classes, along with additional near-boundary samples. In the final stage, non-boundary data are compressed using hierarchical clustering, and cluster centroids are retained. This approach effectively reduces dataset size while preserving classification performance. Shalaby et al.^14^ introduced the density-based border identification (DBI) algorithm, a novel method for reducing the training cost of SVMs by selecting informative border instances. DBI identifies boundary samples likely to serve as support vectors, using a core score analysis for each class of the data. The method is significantly decreasing training time while maintaining classification accuracy. The authors also proposed two variants. Border-biased random instance selection (BRI) retains points that have high probability to lie in the border and the overlapping areas and border-biased random instance selection with eXclusion (BRIX) retains points that have high probability to lie in the border areas of the classes and away from overlapping areas. This is achieved through a computation for a pureness score. Two more methods are proposed. Support vector oracle (SVO) identities the reduced set by computing the support vectors and their k nearest neighbors from the same class in a low dimensional space, where they can be selected in higher dimensions for training and classification. Support Vector Oracle with eXclusion (SVOX) reduces support vector seeds by excluding those with impure neighborhoods below a certain threshold.

Perera-Lago et al.^15^ conducted a comprehensive evaluation of eight data reduction techniques tailored for deep learning, with a focus on reducing both computational cost and carbon footprint. Their work emphasizes the intersection of efficiency and sustainability, in line with Green AI principles. The study examines two datasets collision and dry bean using a MLP classifier. The previous studies have demonstrated that instance selection for data reduction can be utilized to shrink the size of the training dataset while maintaining the performance of classifiers such as SVM and MLP. A common approach in earlier work involves dividing the dataset into clusters and identifying the samples near the decision boundary, usually by detecting clusters that contain data points from multiple classes. However, these strategies are heavily influenced by the dataset’s distribution and the presence of outliers. In addition, they are highly dependent on factors such as the cluster granularity and the initialization of centroids. For example, when clusters are created with high granularity, most of them tend to represent only one class, in contrast, with lower granularity, many clusters will likely include multiple class labels, which reduces the reliability of the selection process. Another limitation is that many of these methods provide little to no control over the final size of the reduced dataset.

Cherrat et al.^16^ proposed a hybrid approach combining K-Means and DBSCAN to improve fingerprint image segmentation. Their method applies image preprocessing filters, then uses K-Means to identify foreground regions, followed by DBSCAN to refine segmentation boundaries. Importantly, their use of clustering is not aimed at improving cluster quality or structure, but rather serves as a tool for enhancing biometric image segmentation. The focus of their work is on image-level processing, not on data reduction or instance selection. Chen et al.^17^ proposed a parameter selection algorithm for DBSCAN based on a two class K-Means clustering. Their method uses K-Means to divide the data and estimate optimal values for $\varepsilon$ and MinPts by analyzing intra and inter cluster distances. The goal is to automate DBSCAN parameter tuning, rather than perform clustering or reduce dataset size. Gholizadeh et al.^18^ introduced K-DBSCAN, an improved DBSCAN algorithm tailored for big data applications. Their method uses K-Means++ to partition large datasets into smaller subsets, and then applies DBSCAN within each partition to accelerate clustering. The overall time complexity of their approach is expressed as $O\big ( (k \cdot n) + \left( \frac{n}{k} \right) ^2)$, which significantly reduces the computational burden compared to the $O(n^2)$ complexity of standard DBSCAN.

In addition to these instance selection techniques and hybrid clustering approaches that combine K-Means and DBSCAN, recent works have explored energy-aware and sustainability-driven solutions aligned with Green AI principles, including efficient learning for smart systems Naz et al.^19^, energy-optimized wireless networks Haseeb et al.^20^, and intelligent feature-driven medical image analysis Saba et al.^21^ These studies highlight the growing importance of computational efficiency in modern AI systems. Unlike these model- and system-level techniques, our method focuses on instance-level data reduction to improve learning efficiency without modifying model architectures.

## Proposed method

The exponential growth of training data in machine learning has created an urgent need for efficient data reduction techniques that can minimize computational costs, maintain model accuracy, and reduce environmental impact. The complementary strengths and limitations of K-Means and DBSCAN clustering motivate our proposed hybrid method that aim to combine the geometric simplicity of K-Means with the density-awareness and noise resilience of DBSCAN. This approach is particularly promising in the context of data reduction and Green AI, where accurate yet efficient clustering is essential.

Our proposed **KM-DBSCAN** framework directly addresses critical challenges in the context of *Green AI* and machine learning:

1. The high computational cost of density-based clustering on large-scale datasets, which typically exhibits $O(n^2)$ complexity.
2. The challenge of overlapping class distributions, which can lead to ambiguous cluster boundaries and reduced classification accuracy.
3. The sensitivity of DBSCAN to parameter selection particularly $\varepsilon$ and *MinPts* which can significantly impact clustering quality if not properly tuned.
4. The need for intelligent data reduction strategies that minimize dataset size without sacrificing model performance.

### An enhanced density-based spatial clustering algorithm (KM-DBSCAN)

Clustering high-dimensional datasets efficiently while preserving the ability to detect arbitrary shapes and outliers remains a significant challenge in unsupervised learning. Traditional DBSCAN, while effective for density-based clustering, suffers from computational inefficiency on large-scale data due to its $O(n^2)$ pairwise distance computations.

To address this limitation, a two-stage hybrid approach is proposed:

1. **K-means preprocessing:** The dataset is first compressed into *k* representative centroids using K-means clustering. The value of *k* is empirically chosen to maintain the overall density structure of the dataset.
2. **DBSCAN clustering:** The resulting centroids are then fed into DBSCAN to perform the density-based clustering.
3. **Labeling data:** Finally labeling data using the DBSCAN labels on KMeans indices.

This method leverages the *O*(*nk*) scalability of K-means to preprocess the data, allowing DBSCAN to operate on a condensed set of centroids, thus significantly reducing runtime without sacrificing accuracy. Let the dataset be defined as $X = \{x_1, x_2,$ $\ldots , x_n\}$. K-means partitions *X* into *k* clusters $C_1, C_2,$ $\ldots , C_k$ with centroids $\mu _1, \mu _2,$ $\ldots , \mu _k$, by minimizing the intra-cluster variance and can calculate centroids using following Eq. (3):

3 $$\begin{aligned} \mu _i = \frac{1}{|C_i|} \sum _{x_j \in C_i} x_j \end{aligned}$$

Unlike prior hybrid approaches, we discard the cluster assignments and retain only the centroids as input for DBSCAN. This reduces the clustering problem from *n* data points to *k* centroids, reducing DBSCAN’s time complexity from $O(n^2)$ to $O(k^2)$, which is significantly lower than the partition-based cost of $O\left( \left( \frac{n}{k}\right) ^2\right)$ used in K-DBSCAN^18^. DBSCAN then processes the centroids using parameters $(\epsilon , \text {MinPts})$. Parameter tuning is inherently simpler at the centroid level due to their more regular spatial distribution. A centroid $\mu _i$ is considered a **core point** if it has at least $\text {MinPts}$ neighboring centroids within $\epsilon$-distance:

4 $$\begin{aligned} \text {Core}(\mu _i) = {\left\{ \begin{array}{ll} \text {True} & \text {if } \big |\{\mu _j \mid \Vert \mu _i - \mu _j\Vert \le \epsilon \}\big | \ge \text {MinPts}, \\ \text {False} & \text {otherwise.} \end{array}\right. } \end{aligned}$$

where $\{\mu _1,$ $\mu _2,$ $\ldots , \mu _k\}$ is the set of centroids, $\Vert \cdot \Vert$ denotes the Euclidean ($L_2$) distance, $\epsilon$ is the neighborhood radius, and $\text {MinPts}$ is the density threshold for core point classification.

**Fig. 2:**
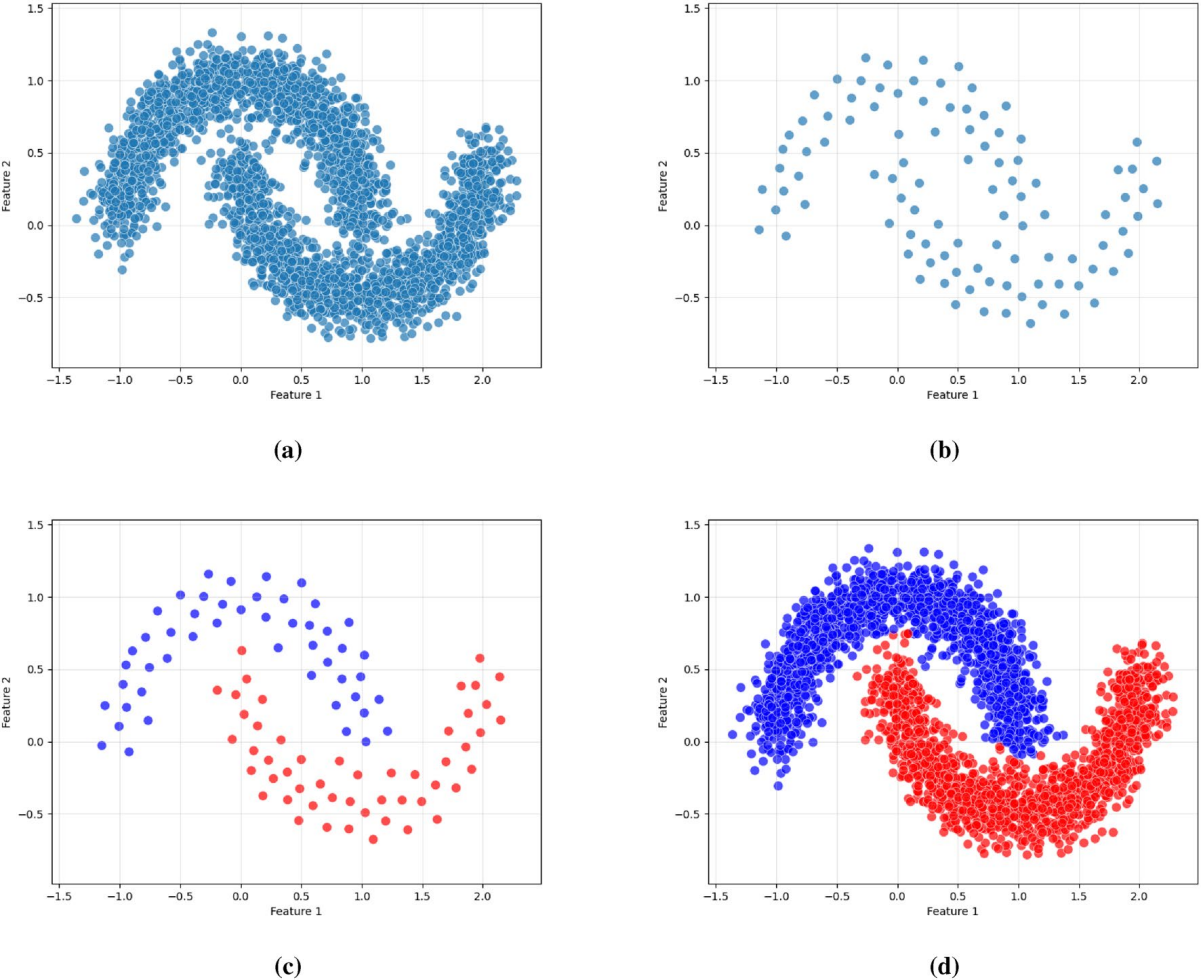
Two moons dataset visualization: (**a**) Initial dataset, (**b**) Centroids Identified by K-means clustering, (**c**) DBSCAN clustering on centroids, and (**d**) Labeling data using centroids.

**Fig. 3:**
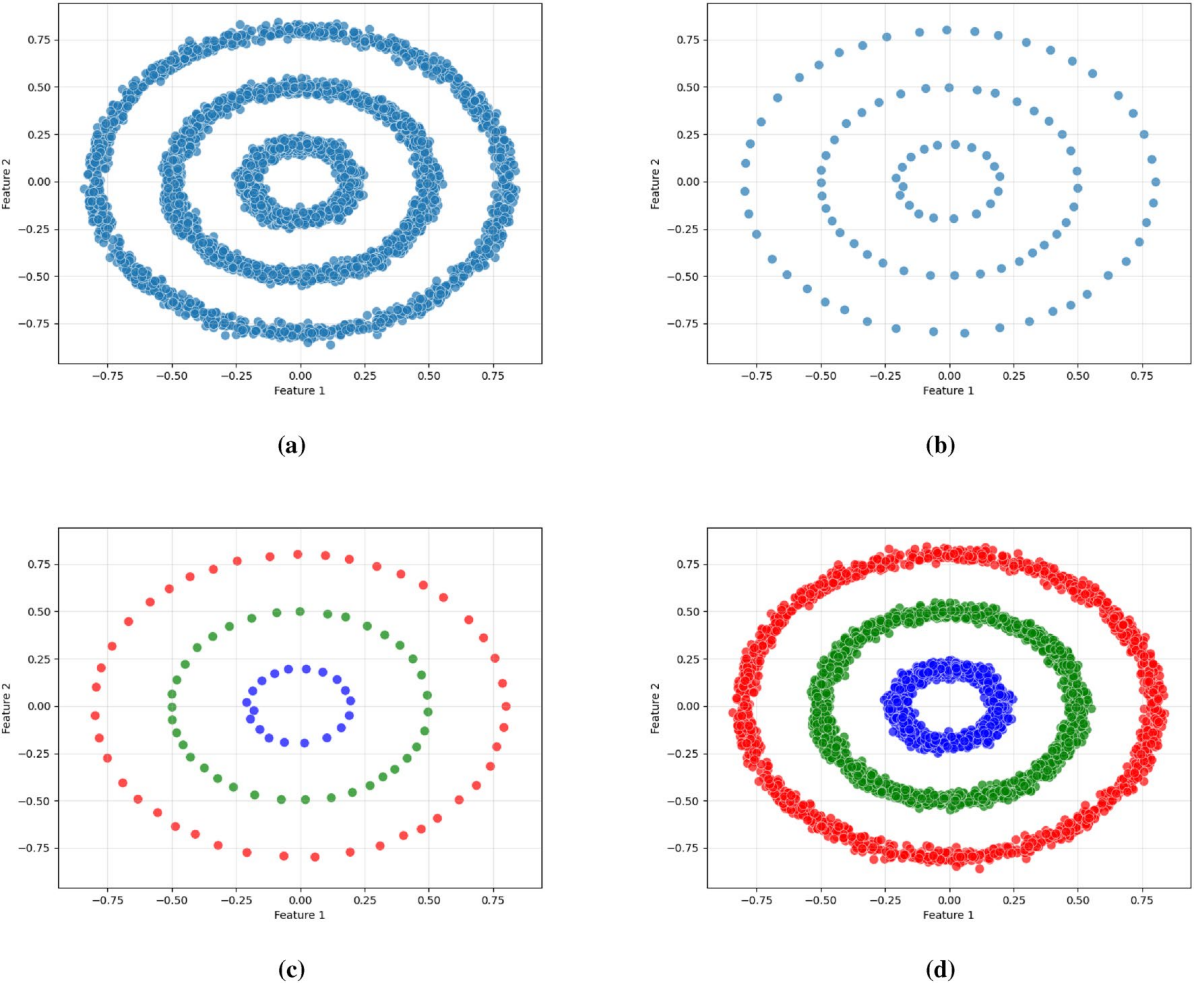
Synthetic concentric ring dataset visualization: (**a**) Initial dataset, (**b**) Centroids identified by K-means clustering, (**c**) DBSCAN clustering on centroids, and (**d**) Labeling data using centroids.

**Fig. 4:**
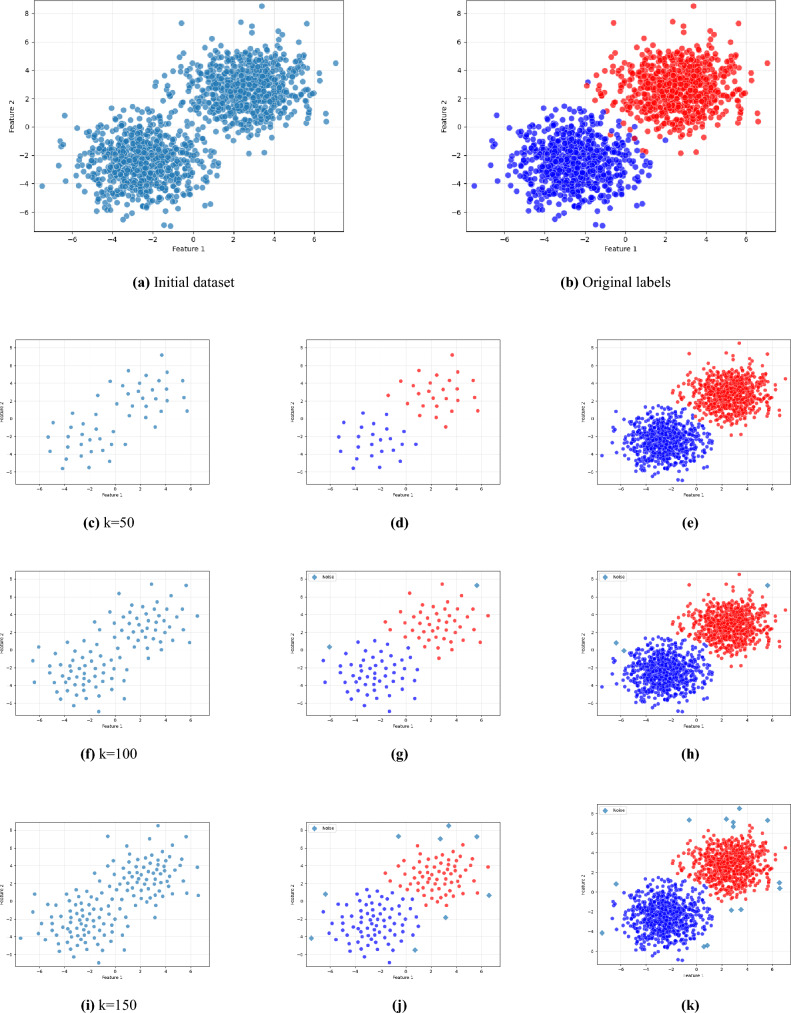
Synthetic overlapped dataset: First column different centroids by K-means, second is DBSCAN clustering on centroids and finally is labeling data using centroids.

As illustrated in Figs. 2, 3, and 4, the proposed framework is demonstrated on three synthetic datasets: First, the Two Moons dataset, a classic example of non-convex, interleaved clusters. Second, a concentric ring dataset Fig. 3, composed of non-overlapping clusters. Third, a Gaussian mixture dataset with partially overlapping distributions Fig. 4. This diverse highlights the geometric flexibility and robustness of KM-DBSCAN across a broad range of clustering scenarios. As shown, the Two Moons and the concentric ring datasets confirm the framework’s ability to manage non-linearly separable structures. In contrast, Fig. 4 focuses on overlapping classes as one of the most challenging clustering scenarios. Traditional DBSCAN often struggles under such conditions, requiring delicate tuning of $\epsilon$ and *MinPts*, and frequently resulting in over-merged clusters or excessive noise labels. Our hybrid approach mitigates these issues through centroid-level preprocessing, which simplifies the density estimation process and improves separation. As demonstrated in Fig. 4, adjusting the number of *k* centroids (not too few to lose structure, nor too many to overfit) enables DBSCAN to capture meaningful densities, even in regions of overlap where the centroids from the different classes are well separated. By clustering a reduced, spatially balanced set of centroids, KM-DBSCAN reduces data complexity while also alleviating parameter sensitivity. After clustering the centroids, labels are propagated back to the original dataset, yielding final assignments that combine the stability of K-means with the geometric adaptability of DBSCAN. This results in improved separation of overlapping clusters and enhanced robustness to noise. Moreover, the proposed KM-DBSCAN framework offers a significant computational advantage. The overall time complexity consists of three components: the K-Means preprocessing phase with complexity *O*(*nk*), the DBSCAN clustering on centroids with complexity $O(k^2)$, and the label propagation step with complexity *O*(*n*). Thus, the total complexity becomes $O(nk + k^2)$, which is substantially more efficient than the original DBSCAN complexity of $O(n^2)$, especially when $k \ll n$. This efficiency gain is critical for large-scale datasets and aligns with the goals of *Green AI* by reducing computational cost and energy consumption. Algorithm 1, represents the proposed KM-DBSCAN clustering method.

**Algorithm 1:**
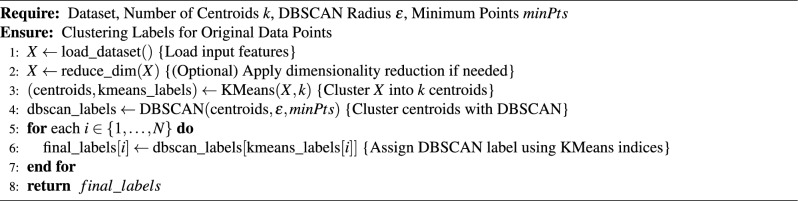
KM-DBSCAN Algorithm

**Algorithm 2:**
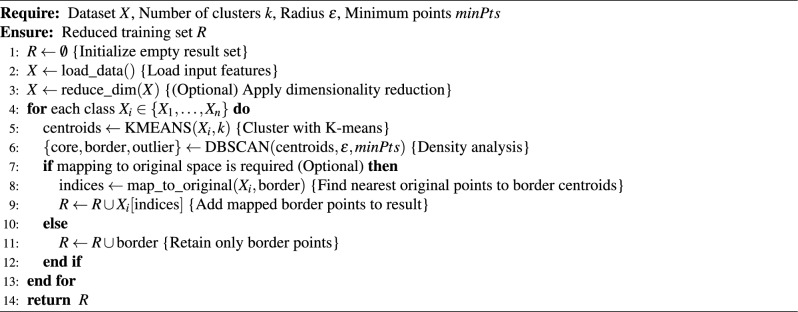
Intelligent data reduction using KM-DBSCAN for border detection

### Intelligent data reduction

**Fig. 5:**
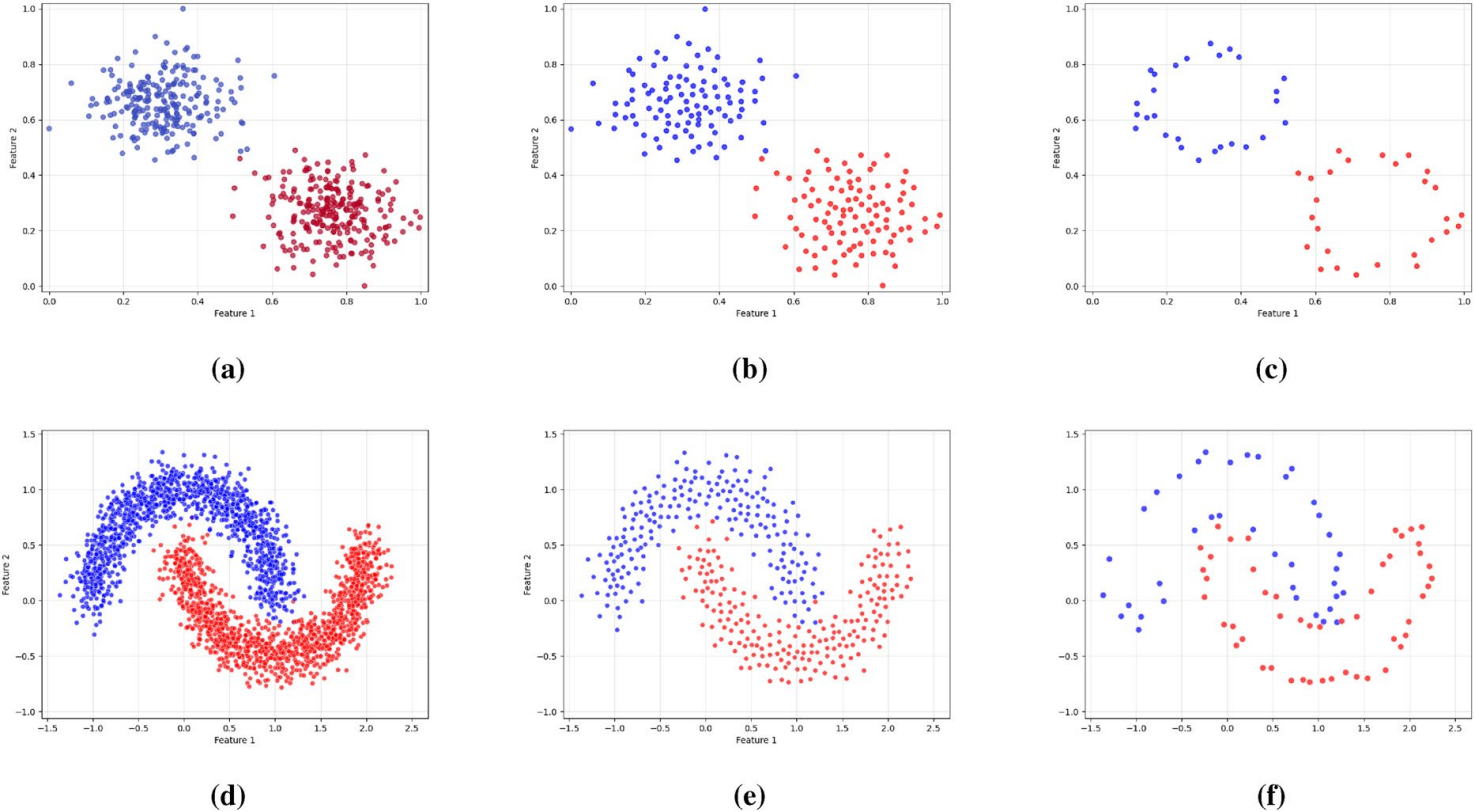
KM-DBSCAN border detection for data reduction across two datasets. The top row shows gaussian mixture, the bottom shows two moons. Columns represent: original data, K-means centroids, and DBSCAN border points.

In this section, a density based method for data reduction is presented using KM-DBSCAN algorithm, where the data points of each class are processed independently. Firstly using k-means algorithm to generate *k* centroids for each class separately. These centroids provide a condensed abstraction of the class distribution, which helps to reduce the complexity of the original dataset. Importantly, this step also leads to a reduction in the overlap between data points, as evidenced by the outcome shown in Fig. 5b. Then using centroids data to extract border points using the DBSACN algorithm on each class separately and this step is an enhancement for the DBSCAN performance to detect border points with determine perfect parameters like epsilon and min-points and can represent this mathematic in general rule as:

5 $$\begin{aligned} \mathscr {R} = \bigcup _{i=1}^{|\mathscr {Y}|} \text {RetainBorder}(\text {DBSCAN}(\text {KMEANS}(X_i, k_i), \epsilon , \text {minPts}) \end{aligned}$$

where $\mathscr {Y}$ is the set of all class labels in the dataset, $X_i \subset X$ represents samples from class $i \in \mathscr {Y}$, $k_i$ = $\lceil \alpha$ $|X_i| \rceil$ is the class specific cluster count where $\alpha$ is percentage number from $|X_i|$ and $\text {RetainBorder}()$ extracts the border points from DBSCAN results. The parameters $\epsilon$ and $\text {minPts}$ are used to classify each centroid $\mu _i$, which belongs to the set of all centroids produced by K-means, into one of three types based on the DBSCAN criteria:

6 $$\begin{aligned} \text {Type}(\mu _i) = {\left\{ \begin{array}{ll} \text {Core} & \text {if } |N_\epsilon (\mu _i)| \ge \text {minPts} \\ \text {Border} & \text {if } \exists \mu _j \in N_\epsilon (\mu _i) \text { such that } \text {Type}(\mu _j) = \text {Core} \\ \text {Noise} & \text {otherwise} \end{array}\right. } \end{aligned}$$

where $N_\epsilon (\mu _i)$ = $\{\mu _j$ $\in$ $\mathscr {C}$ $\mid \Vert \mu _i$ - $\mu _j\Vert$ $\le \epsilon \}$ is the set of neighbors of $\mu _i$ within radius $\epsilon$. Border points are preserved as the reduced set for training a machine learning model where outliers and core points are eliminated as they usually far from the region of decision boundaries, as shown in Fig. 5c. where, The user defined parameters (*k*,$\epsilon$,$\text {MinPts}$) control the reduction ratio of the data. For high-dimensional datasets, we applied PCA or UMAP for features extraction and dimensionality reduction as a preprocessing step to reduce the effect of the curse of dimensionality. KMEANS and DBSCAN algorithms are preferred to operate in low dimensions where their performance usually drops in high dimensions as the distance measures between objects start to be less reliable^22^. After identifying the indices of the reduced set in a low dimensional space, it can be mapped back to the original space or any other higher dimensions for the training and classification purposes towards optimizing the performance measures according to the machine learning model that is used. As in line with GreenAI, applying dimensionality reduction as a preprocessing step is considered beneficial for data reduction, as it enables more efficient repetitive training and decreases the overall computational cost.

7 $$\begin{aligned} X' = \text {UMAP}(X, d') \quad \text {or} \quad X' = \text {PCA}(X, d') \end{aligned}$$

**Fig. 6:**
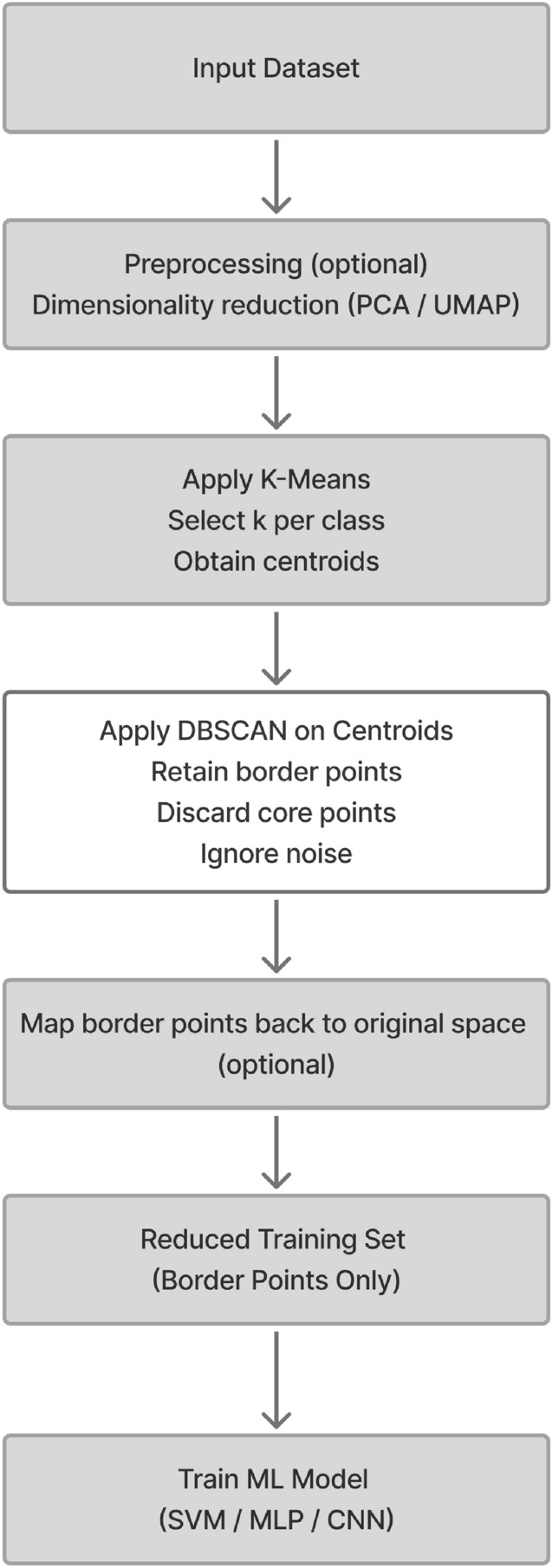
Overview of the proposed KM-DBSCAN data reduction framework.

Where *X* represent original data and $d'$ number of features, but in sometime we need to map border points to the original dimension to train a model like Convolutional Neural Networks. Algorithm 2 represents a pseudo-code for the proposed Intelligent Data Reduction. For better illustration of the workflow, the corresponding flowchart is presented in Fig. 6.

**Fig. 7:**
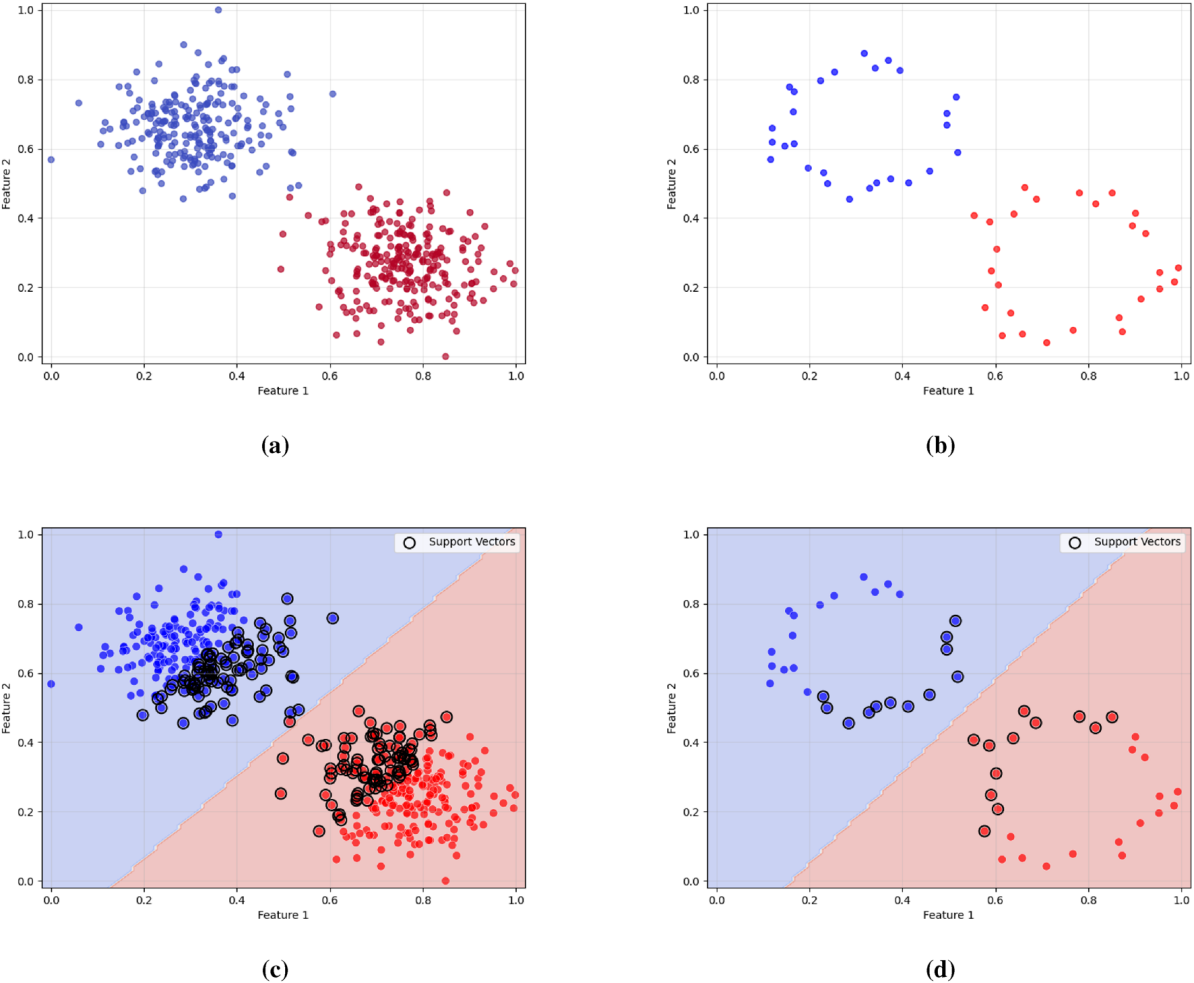
SVM decision boundaries on synthetic blobs: (**a**) Original data, (**b**) KM-DBSCAN reduced data, (**c**) SVM on full data, (**d**) SVM on reduced data. Support vectors marked with black borders.

**Fig. 8:**
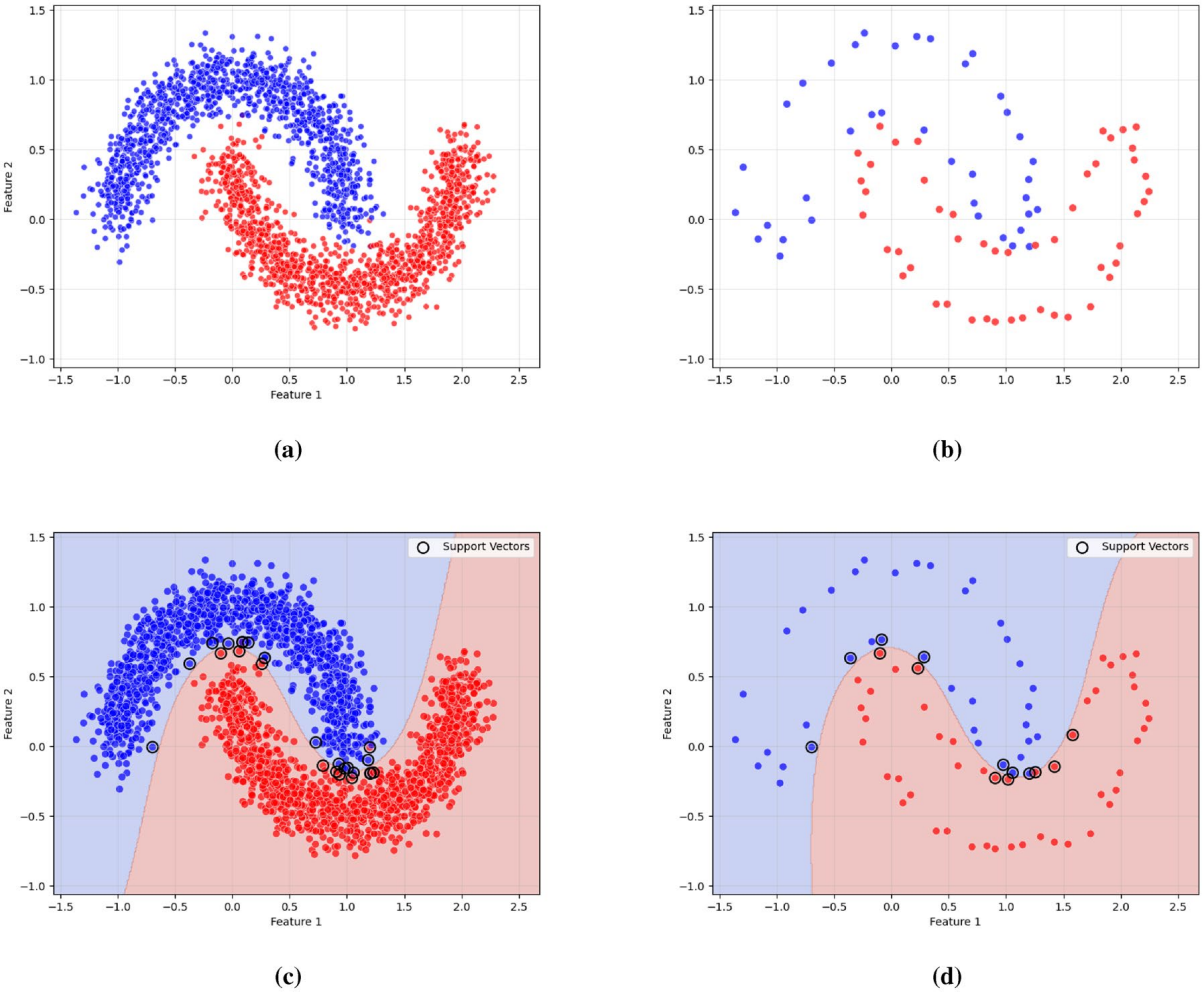
SVM decision boundaries on two moons: (**a**) Original data, (**b**) KM-DBSCAN reduced data, (**c**) SVM on full data, (**d**) SVM on reduced data. Support vectors marked with black borders.

Figures 7 and 8 illustrate the effectiveness of the proposed KM-DBSCAN approach in preserving classification quality, even after substantial data reduction. In both synthetic blobs dataset Fig. 7 and the two moons dataset Fig. 8. Subfigures (a) and (b) show the original and reduced datasets, respectively. Subfigures (c) and (d) compare the SVM decision boundaries trained on the full dataset and on the reduced dataset. As observed, the reduced data obtained using KM-DBSCAN (subfigures (b) and (d)) successfully preserves the structure of the original classes, allowing the SVM classifier to maintain a clear and accurate separation boundary. Even with a drastic reduction in the number of training points, the decision boundaries remain closely aligned with those learned on the original dataset. These visualizations demonstrate that our method can significantly reduce data size while retaining critical class distribution characteristics, which is essential in large-scale or real-time medical applications. The effectiveness of our approach is validated by the significant improvements observed in the next section.

## Experimental results

This section present the experimental setup along with the results obtained from applying the proposed KM-DBSCAN method to several benchmark datasets. The experiments evaluate the improvement in the performance of our approach in clustering and data reduction while maintaining high classification accuracy.All experiments were conducted in Python 3.10.4 on a local workstation equipped with an Intel Core i7-10750H CPU, an NVIDIA GeForce GTX 1660 Ti GPU, and 16 GB RAM, running Windows 10. The implementation utilizes the scikitlearn^23^ and codecarbon^24^ libraries. Carbon emissions were monitored using the CodeCarbon tracker (v2.3.5) in machine-mode, which records real-time CPU/GPU power consumption and estimates emissions in kgCO_2_eq. The default IEA regional carbon-intensity factor for the Egypt/North Africa region was applied. To ensure accurate measurement, the tracker was activated only during the classifier training phase and stopped immediately upon completion, thereby accounting exclusively for energy consumption associated with model training and excluding idle or post-processing overhead. CodeCarbon’s default hardware-power profiles were used, and cloud-mode was disabled since all experiments were executed locally. This configuration ensures transparent and reproducible energy and emission estimation.

### Datasets

The KM-DBSCAN algorithm has been evaluated on synthetic and real datasets, which are described as follows:

**Banana:** It is a two-dimensional dataset, for two classes with a degree of overlap and a total of 5,300 samples. In our experiments, 4,240 samples are allocated for training, while 1,060 samples are reserved for testing^25^.

**USPS:** It is composed of 16x16 grayscale images for handwritten digits in 10 classes automatically scanned from envelopes by the U.S. Postal Service^26^. The data is split into 7,291 samples for training and 2,007 for testing.

**Adult9a:** It contains 32,561 training samples and 16,281 testing samples. Each instance corresponds to an individual described by 123 attributes. The task is framed as a binary classification problem to predict if the annual income of an individual exceeds $50,000. Of the total, 24,720 samples belong to the negative class and 7,841 to the positive class, indicating a strong class imbalance. Preprocessing steps include removing instances with missing values and applying one-hot encoding to categorical attributes^27^.

**Collision:** Serves as a benchmark for evaluating data reduction methods in safety-critical mobility applications, where computational efficiency and predictive accuracy are paramount. This binary classification dataset predicts whether a platoon of vehicles will collide, based on 25 numerical features such as vehicle count, speed, and kinematic parameters. It contains 107,210 samples, with 69,348 labeled as collisions (positive class) and 37,862 as non-collisions (negative class), resulting in a moderately imbalanced class distribution. This dataset is employed to evaluate data reduction methods aimed at minimizing resource consumption in safety-critical mobility applications^28^

**DryBean:** A multi-class classification dataset consisting of 13,611 samples representing seven different types of dry beans, each described by 16 geometric features extracted from images, such as area, perimeter, and eccentricity. The class distribution is unbalanced, with Barbunya (1,322), Bombay (522), Cali (1,630), Dermason (3,546), Horoz (1,928), Seker (2,027), and Sira (2,636) samples. For experimental purposes, the classes are encoded from 0 to 6 following this order. The dataset is used to evaluate data reduction methods in classification tasks involving multiple and imbalanced classes^29,30^ .

**Melanoma skin cancer:** An image classification dataset designed for detecting melanoma skin cancer from non-dermoscopic images. It comprises 10,605 images, with 9,605 allocated for training and 1,000 for evaluation. The dataset includes two classes: malignant and benign lesions. All images are standardized to have 300 pixels on their longest side. Due to challenges in medical image collection and expert annotation, the dataset is relatively small and reflects real-world constraints. It is utilized to evaluate model performance under limited data conditions in medical image analysis^31^.

### Metrics

To assess the effectiveness of the proposed KM-DBSCAN method, we employ multiple evaluation metrics targeting predictive accuracy, clustering quality, data reduction efficiency, performance speedup and computational gain.

**Classification accuracy.** The predictive accuracy of the classifier trained using the reduced dataset produced by KM-DBSCAN is evaluated using classification accuracy, defined in Equation (8):

8 $$\begin{aligned} \text {Accuracy} = \frac{TP + TN}{TP + TN + FP + FN} \end{aligned}$$

where *TP* denotes the number of true positives, *TN* the number of true negatives, *FP* the number of false positives, and *FN* the number of false negatives. This metric indicates the ratio of correctly classified instances to the total number of predictions.

**Adjusted rand index (ARI).** To measure clustering quality, we use the Adjusted Rand Index (ARI), which ranges from -1 (worst case) to 1 (perfect case), with 0 indicating random labeling. ARI measures the similarity between the clustering result $\mathscr {C}$ and the ground-truth labels $\mathscr {C}^*$. It is calculated as:

9 $$\begin{aligned} \text {ARI}(\mathscr {C}, \mathscr {C}^*) = \frac{ \sum _{i,j} \left( {\begin{array}{c}n_{ij}\\ 2\end{array}}\right) - \frac{ \left[ \sum _i \left( {\begin{array}{c}a_i\\ 2\end{array}}\right) \sum _j \left( {\begin{array}{c}b_j\\ 2\end{array}}\right) \right] }{ \left( {\begin{array}{c}n\\ 2\end{array}}\right) } }{ \frac{1}{2}\left[ \sum _i \left( {\begin{array}{c}a_i\\ 2\end{array}}\right) + \sum _j \left( {\begin{array}{c}b_j\\ 2\end{array}}\right) \right] - \frac{ \left[ \sum _i \left( {\begin{array}{c}a_i\\ 2\end{array}}\right) \sum _j \left( {\begin{array}{c}b_j\\ 2\end{array}}\right) \right] }{ \left( {\begin{array}{c}n\\ 2\end{array}}\right) } } \end{aligned}$$

Here, $n_{ij}$ denotes the number of common elements between cluster $i \in \mathscr {C}$ and class $j \in \mathscr {C}^*$, $a_i = |\mathscr {C}_i|$ is the size of predicted cluster *i*, $b_j = |\mathscr {C}^*_j|$ is the size of ground truth class *j*, and *n* is the total number of samples.

**Reduction ratio.** The data reduction efficiency is evaluated using the Reduction Ratio (*R*), which represents the fraction of retained data points after reduction:

10 $$\begin{aligned} R = \frac{|\mathscr {X}'|}{|\mathscr {X}|} \quad \text {where} \quad {\left\{ \begin{array}{ll} \mathscr {X} & \text {= Original dataset (size } N\text {)} \\ \mathscr {X}' & \text {= Reduced dataset (size } N'\text {)} \\ \end{array}\right. } \end{aligned}$$

**Speedup factor.** The improvement in computational performance is measured using the Speedup Factor (*S*):

11 $$\begin{aligned} S = \frac{T_{\text {original}}}{T_{\text {reduced}}} \end{aligned}$$

where $T_{\text {original}}$ is the execution time using the full dataset in original scenario, and $T_{\text {reduced}}$ is the time after applying KM-DBSCAN.

**Carbon emission.** The carbon emissions were estimated using the CodeCarbon tracker based on the following formula:

12 $$\begin{aligned} \text {CO}_{2} \text {Emissions} = \text {Energy Consumed (kWh)} \times \text {Carbon Intensity (gCO}_{2}\text {/kWh)} \end{aligned}$$

where energy consumption is derived from hardware power profiles and runtime duration.

### KM-DBSCAN for data clustering

The KM-DBSCAN method consistently achieves high clustering quality while substantially reducing computational cost across all evaluated datasets. The Adjusted Rand Index (ARI), which ranges from -1 (worst) to 1 (perfect agreement), with 0 indicating random labeling, is used to measure alignment with ground-truth labels. Across experiments, KM-DBSCAN demonstrates strong and often near-perfect ARI scores, particularly on simpler datasets. However, performance remains influenced by dataset characteristics and parameter sensitivity. By operating on K-means centroids rather than the full dataset, the method maintains high clustering accuracy, as shown in Table 1. In the Two Moons dataset, both standard DBSCAN and KM-DBSCAN achieve an ARI of 0.976 however, KM-DBSCAN uses only 100 representative points instead of the full 3,000, resulting in a 0.0333 reduction ratio and a 23.9$\times$ speedup. Similarly, in the Concentric Ring dataset, the ARI remains at 1.000, with only 100 points compared to 4,600 in the original dataset, achieving a 0.0217 reduction ratio and a 23.55$\times$ speedup.

In real-world datasets, KM-DBSCAN preserves or even improves clustering quality while dramatically reducing runtime. For the USPS dataset (7,291 samples), dimensionality was first reduced from 256 to 64 using UMAP before clustering. Traditional DBSCAN takes 0.2825 s with an ARI of 0.941. In contrast, KM-DBSCAN, using only 500 samples with a 0.068 reduction ratio, runs in just 0.0049 s achieving a 57.65$\times$ speedup and slightly improves the ARI to 0.942. For the Dry Bean dataset (13,611 samples), dimensionality was reduced from 16 to 10 using UMAP. Traditional DBSCAN achieves an ARI of 0.5818 in 0.4750 s. In contrast, KM-DBSCAN, using only 500 samples with a 0.036 reduction ratio, reduces the runtime to 0.0059 s making it 80.50$\times$ faster while increasing the ARI to 0.697.

To assess the stability and reliability of the proposed KM-DBSCAN clustering, each experiment was repeated five times using different random seeds. The average ARI and the corresponding standard deviation across runs are reported in Table 2. In addition, the runtime was also averaged over the five runs, and its mean ± standard deviation were computed to evaluate the consistency of computational efficiency. As shown, the results exhibit high mean accuracy with consistently low variance across all datasets, indicating strong robustness, runtime stability, and insensitivity to random initialization.

**Table 1:** Performance comparison of KM-DBSCAN versus original DBSCAN on synthetic and real datasets.

Dataset	Data size	DBSCAN Exe Time (s)	ARI DBSCAN	K	Reduction Ratio	KM-DBSCAN Exe Time (s)	ARI KM-DBSCAN	Speedup
Two Moons	3000	0.0239	0.976	100	0.0333	0.0010	0.976	23.90
Concentric ring	4600	0.0471	1.000	100	0.0217	0.0020	1.000	23.55
USPS	7291	0.2825	0.941	500	0.0680	0.0049	0.942	57.65
Dry bean	13611	0.4750	0.5818	500	0.0360	0.0059	0.697	80.50

**Table 2:** ARI mean ± Std over multiple runs for KM-DBSCAN clustering model.

Dataset	ARI mean ± Std	Runtime (s) Mean ± Std
Two moons	$0.9673 \pm 0.0131$	$0.0022 \pm 0.0004$
Concentric ring	$1.000 \pm 0.000$	$0.0026 \pm 0.0005$
USPS	$0.9398 \pm 0.0025$	$0.0060 \pm 0.0006$
Dry bean	$0.6904 \pm 0.0051$	$0.0110 \pm 0.0031$

Our approach directly addresses two critical limitations of traditional DBSCAN: (1) the well-known difficulty in selecting optimal density parameters (i.e., eps and $\text {MinPts}$), and (2) the algorithm’s sensitivity to overlapping data structures. As illustrated in Table 3, our method reframes this challenge into a more controllable centroid selection task. By using a reduced number of centroids (e.g., $k = 50$), we simplify the parameter tuning process while achieving excellent performance (ARI = 0.979 with 0 noise points) compared to traditional DBSCAN (ARI = 0.960 with 10 noise points). In contrast, traditional DBSCAN exhibits unstable behavior, where minor parameter changes can lead to drastically different clustering results. Although increasing the number of centroids (e.g., $k = 150$) introduces a moderate increase in complexity (14 noise points vs. 0 at $k = 50$), our method still maintains high accuracy (ARI = 0.953) and fast execution (0.003 s). This demonstrates graceful degradation, unlike DBSCAN’s abrupt failure modes. Interestingly, we observe an inverse relationship between the number of centroids and sensitivity to parameter tuning: lower values of *k* (such as $k = 50$) require less tuning effort than higher values (e.g., $k = 150$). This trade-off highlights a key advantage of our method, allowing users to strategically balance clustering precision and tuning complexity by adjusting centroid density a flexibility fundamentally lacking in standard DBSCAN.

These results clearly demonstrate that KM-DBSCAN preserves clustering quality while using only a fraction of the original data and cutting computation time. This combination of accuracy and efficiency makes it particularly suitable for large scale or resource constrained clustering tasks. To further support these observations, we conducted a systematic parameter sensitivity analysis across multiple configurations of $\varepsilon$, *MinPts*, and centroid count *k*. Table 4 presents the results averaged over seven runs per setting. The findings confirm that KM-DBSCAN consistently achieves high ARI scores and low computation time across a wide range of parameter values, while DBSCAN exhibits greater volatility. This empirical evidence reinforces our claim that KM-DBSCAN offers a more stable and tunable clustering framework.

**Table 3:** Performance comparison of KM-DBSCAN on gaussian mixture dataset with different number of centroids.

	Reduction Ratio	Execution Time (s)	ARI	Speedup	Noise
DBSCAN (whole data set = 1500)	–	0.021472	0.960	–	10
KM-DBSCAN (k=50)	0.03	0.0009742	0.979	22.04	0
KM-DBSCAN (k=100)	0.06	0.001951	0.967	11.00	3
KM-DBSCAN (k=150)	0.10	0.0029271	0.953	7.33	14

**Table 4:** Comparison between DBSCAN and KM-DBSCAN across multiple parameter settings.

Method	$\varepsilon$	MinPts	ARI	Time (s)
	2.75	399	0.91	0.0249
	2.76	399	0.00	0.0240
	2.75	405	0.913	0.0229
DBSCAN (whole data set = 1500)	2.77	405	0	0.0269
	2.77	406	0	0.0229
	2.80	407	0	0.0249
	2.80	415	0.902	0.0229
	1.97	2	0.979	0.001
	1.97	3	0.979	0.001
	1.98	3	0.979	0.001
KM-DBSCAN (k=50)	1.98	2	0.979	0.001
	1.99	2	0.979	0.001
	2.00	4	0.979	0.001
	2.00	5	0.979	0.001
	2.30	14	0.951	0.001
	2.31	14	0.955	0.001
	2.31	15	0.964	0.001
KM-DBSCAN (k=100)	2.33	14	0.967	0.001
	2.34	14	0.967	0.001
	2.34	16	0.958	0.001
	2.35	16	0.958	0.001
	2.60	36	0.940	0.001
	2.61	36	0.940	0.001
	2.62	35	0.945	0.001
KM-DBSCAN (k=150)	2.63	35	0.945	0.001
	2.64	35	0.953	0.001
	2.64	36	0.943	0.001
	2.63	37	0.938	0.001

### KM-DBSCAN for intelligent data reduction on SVMs

**Fig. 9:**
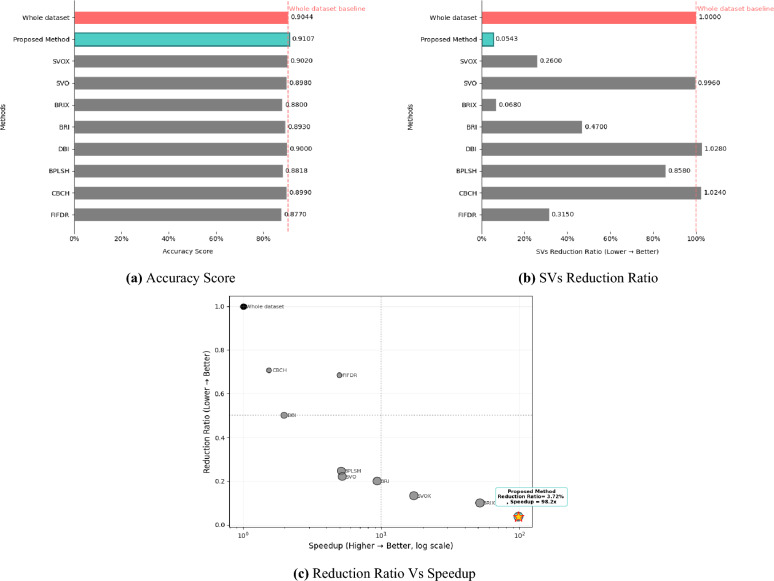
Visualization of banana dataset experimental results.

The SVM classifier was trained with the RBF kernel. The regularization hyper-parameter C and the kernel coefficient $\gamma$ for each classifier were tuned separately by applying a grid search approach. C was selected from {0.01, 1, 10, 100} . while $\gamma$ was selected from {0.001, 0.01, 0.1, 1, ‘scale’, ‘auto’}. Optimization was performed via 5-fold cross-validation during classifier training on the complete dataset. For the multidimensional datasets, UMAP and PCA were employed for feature extraction and dimensionality reduction. For the USPS dataset, UMAP was employed to reduce the data to 64 dimensions, which were used both to train the SVM classifier and to apply data reduction using KM-DBSCAN. For the Adult9a dataset, PCA was employed to reduce to 72 dimensions to train the SVM classifier and to apply data reduction using KM-DBSCAN. The experimental results on the Banana dataset, as presented in Table 5, demonstrate significant improvements achieved by the proposed method compared to existing techniques. Notably, our approach achieves the highest speedup (98.2$\times$) while maintaining competitive accuracy (0.91074), which slightly surpasses the whole-dataset baseline (0.9044). Furthermore, it achieves the lowest reduction ratio (0.0372) and the smallest number of support vectors (61 Vs 1122 whole dataset), corresponding to an SVs reduction ratio of just 0.05436 indicating highly efficient data representation. In contrast, alternative methods either sacrifice accuracy (e.g., FIFDR , BPLSH ) or fail to match the proposed method’s speedup and reduction efficiency (e.g., BRIX , SVOX ). Our approach also records the fastest time 0.0010 s compared to whole dataset 0.0982 s. These results highlight the method’s ability to balance accuracy, computational efficiency, and model compactness, making it a robust solution for large-scale datasets (as shown in Fig. 9). The KM-DBSCAN hyperparameters for this dataset were configured as follows: $k = 200$. DBSCAN parameters were tuned separately for each class, with $\varepsilon = 0.36,\, \text {MinPts} = 12$ for class -1 and $\varepsilon = 0.228,\, \text {MinPts} = 4$ for class 1. The value of $k$ was selected based on the target reduction ratio, while DBSCAN hyperparameters were optimized through centroid-distribution empirical analysis to ensure appropriate density estimation and border-point identification. For the whole dataset, the SVM model was trained with a regularization hyperparameter of $C = 1$ and $\gamma = \text {''scale''}$, whereas the proposed method was trained with $C = 100$ and $\gamma = \text {''scale''}$.

**Table 5:** Results of the KM-DBSCAN data reduction compared to other methods from the related work on the banana dataset.

Method	Accuracy	Loss/Gain in Accuracy	Reduction Ratio	SVs Reduction ratio	Speedup
Whole dataset	0.9044	–	–	–	–
FIFDR^9^	0.8770	− 0.0274	0.684	0.315	4.990
CBCH^10^	0.8990	− 0.0054	0.706	1.024	1.541
BPLSH^11^	0.8818	− 0.0226	0.247	0.858	5.139
DBI^14^	0.9000	− 0.0044	0.500	1.028	1.980
BRI^14^	0.8930	− 0.0114	0.200	0.470	9.360
BRIX^14^	0.8800	− 0.0244	0.100	0.068	51.620
SVO^14^	0.8980	− 0.0064	0.221	0.996	5.230
SVOX^14^	0.9020	− 0.0024	0.133	0.260	17.140
Proposed method	0.91074	+ 0.00634	0.0372	0.05436	98.2

**Fig. 10:**
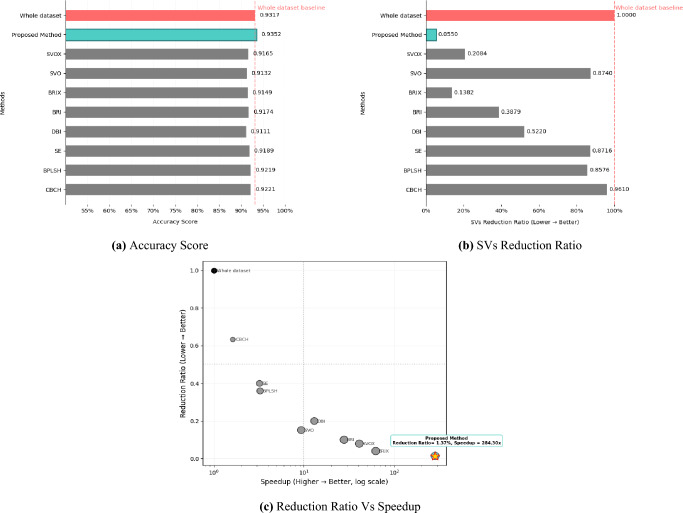
Visualization of USPS dataset experimental results.

As evidenced by the results in Table 6, the proposed method establishes performance on the USPS dataset across all key metrics. Most notably, our approach achieves the highest speedup (284.3$\times$) while maintaining competitive accuracy (0.93522), which slightly surpasses the whole-dataset baseline (0.93173). Furthermore, it achieves the lowest reduction ratio (0.0137) and the smallest number of support vectors (45 Vs 817 whole dataset), corresponding to an SVs reduction ratio of just 0.0550 indicating highly efficient data representation. Furthermore, the achieved 284.3$\times$ speedup and 0.0010 s compared to whole dataset 0.2843 s. time significantly outperform even the fastest existing approaches, including BRIX (62.2$\times$ speedup) and DBI (13.0$\times$ speedup). This combination of improved accuracy, extreme computational efficiency, and model compactness represents a breakthrough in SVM optimization, effectively overcoming the traditional trade-offs between performance and efficiency in machine learning systems (as shown in Fig. 10). The KM-DBSCAN hyperparameters for this dataset were configured as follows: $k = 200$. DBSCAN parameters were tuned separately for each class, with $\varepsilon = 0.57$ and $\text {MinPts} = 13$ for all classes. For the full dataset, the SVM model was trained with a regularization hyperparameter of C = 10 and $\gamma$ = 1, whereas the proposed method was trained with C = 100 and $\gamma$ = 0.1.

**Table 6:** Results of the KM-DBSCAN data reduction compared to other methods from the related work on the USPS dataset.

Method	Accuracy	Loss/Gain in Accuracy	Reduction Ratio	SVs Reduction ratio	Speedup
Whole dataset	0.93173	–	–	–	–
CBCH^10^	0.92207	− 0.00966	0.6318	0.9610	1.6215
BPLSH^11^	0.92192	− 0.00981	0.3600	0.8576	3.2541
Shell Extraction (SE)^12^	0.91893	− 0.0128	0.4000	0.8716	3.2094
DBI^14^	0.91106	− 0.02067	0.2000	0.5220	13.0342
BRI^14^	0.91738	− 0.01435	0.1000	0.3879	27.7140
BRIX^14^	0.91489	− 0.01684	0.0400	0.1382	62.1728
SVO^14^	0.91316	− 0.01857	0.1516	0.8740	9.3301
SVOX^14^	0.91654	− 0.01519	0.0792	0.2084	40.8657
Proposed method	0.93522	+ 0.00349	0.0137	0.0550	284.3000

**Fig. 11:**
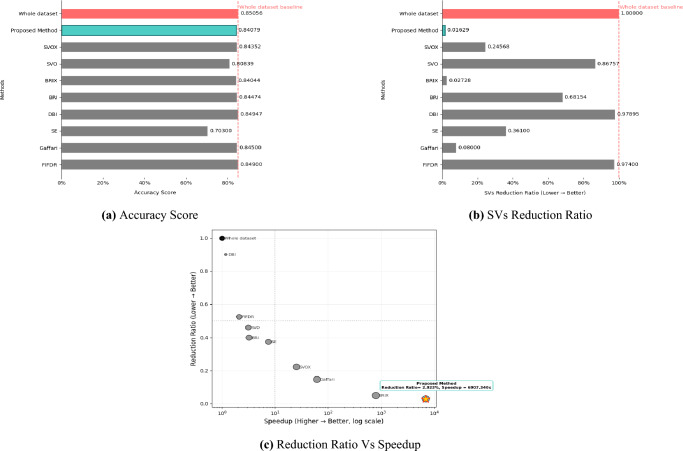
Visualization of Adult9a dataset experimental results.

To evaluate our method on imbalanced datasets, we assess its performance on the challenging Adult9a dataset, with results presented in Table 7. The proposed method demonstrates exceptional robustness to class imbalance, achieving a 6907$\times$ speedup higher than the previous best (BRIX : 790$\times$) while reducing the dataset to only 2.9% of its original size. Despite the dataset’s imbalance, our approach maintains competitive accuracy with a minimal drop of 0.00977 compared to the full dataset baseline (0.84079 vs. 0.85056), substantially outperforming methods such as SVO (0.04217 Loss in Accuracy) and Shell Extraction (0.14756 Loss in Accuracy). The method’s remarkable efficiency is further highlighted by its use of only 192 support vectors compared to whole dataset 11784 SVs (SVs Reduction Ratio = 0.01629). Moreover, it achieves real-time in just 0.009 seconds, compared to the 62.166 seconds runtime of the training dataset model. These results confirm the method’s effectiveness for imbalanced, large-scale problems where both computational efficiency and accuracy retention are critical. The KM-DBSCAN hyperparameters for this dataset were configured as follows: $k = 1000$ for class -1 and $k = 400$ for class 1. DBSCAN parameters were tuned separately for each class, with $\varepsilon = 2.79,\, \text {MinPts} = 313$ for class -1 and $\varepsilon = 1.917,\, \text {MinPts} = 21$ for class 1. For the full dataset, the SVM model was trained with a regularization hyperparameter of C = 1 and $\gamma$ = ”scale”, whereas the proposed method was trained with C = 10 and $\gamma$ = ”auto”. Figure 11 provides a visual representation of these results.

**Table 7:** Results of the KM-DBSCAN data reduction compared to other methods from the related work on the Adult9a dataset.

Method	Accuracy	Loss/Gain in Accuracy	Reduction Ratio	SVs Reduction Ratio	Speedup
Whole dataset	0.85056	–	–	–	–
FIFDR^9^	0.84900	− 0.00156	0.5230	0.97400	2.100
Gaffari^13^	0.84500	− 0.00556	0.1470	0.08000	61.890
Shell Extraction (SE)^12^	0.70300	− 0.14756	0.3740	0.36100	7.500
DBI^14^	0.84947	− 0.00109	0.9000	0.97895	1.1748
BRI^14^	0.84474	− 0.00582	0.4000	0.68154	3.2225
BRIX^14^	0.84044	− 0.01012	0.0500	0.02728	790.5368
SVO^14^	0.80839	− 0.04217	0.4592	0.86757	3.13133
SVOX^14^	0.84352	− 0.00704	0.2230	0.24568	25.380
Proposed method	0.84079	− 0.00977	0.02923	0.01629	6907.340

### KM-DBSCAN for intelligent data reduction on MLP

For the tabular datasets, we followed the data reduction framework described in Perera-Lago et al. To enhance sustainability. Two datasets were used the Collision dataset for binary classification and the Dry Bean dataset for multi-class classification. Before training the model, different statistical and clustering-based reduction methods were applied to minimize dataset size while retaining representative samples. We employed the architecture composed of 10 sequential layers as following dimensions:

$$X \xrightarrow {f_1} \mathbb {R}^{50} \xrightarrow {f_2} \mathbb {R}^{45} \xrightarrow {f_3} \mathbb {R}^{40} \xrightarrow {f_4} \mathbb {R}^{35} \xrightarrow {f_5} \mathbb {R}^{30} \xrightarrow {f_6} \mathbb {R}^{25} \xrightarrow {f_7} \mathbb {R}^{20} \xrightarrow {f_8} \mathbb {R}^{15} \xrightarrow {f_9} \mathbb {R}^{10} \xrightarrow {f_{10}} O$$

All intermediate layers use the rectified linear unit (ReLU) activation function and apply dropout for regularization. The dropout rate was set to 0.50 for the Collision dataset and 0.25 for the Dry Bean dataset. For the Collision dataset, the output layer contains a single neuron with a sigmoid activation, suitable for binary classification. The model was trained using Binary Cross Entropy as the loss function. For the Dry Bean dataset, the output layer consists of seven neurons (one per class) with a softmax activation to produce class probabilities. The model was trained with Categorical Cross Entropy loss, incorporating class weights to balance the dataset and reduce bias toward majority classes. In both experiments, the Adam optimizer^32^ was used with a learning rate of 0.001 and default parameter settings. The Collision dataset model was trained for 600 epochs with a batch size of 1024.

**Fig. 12:**
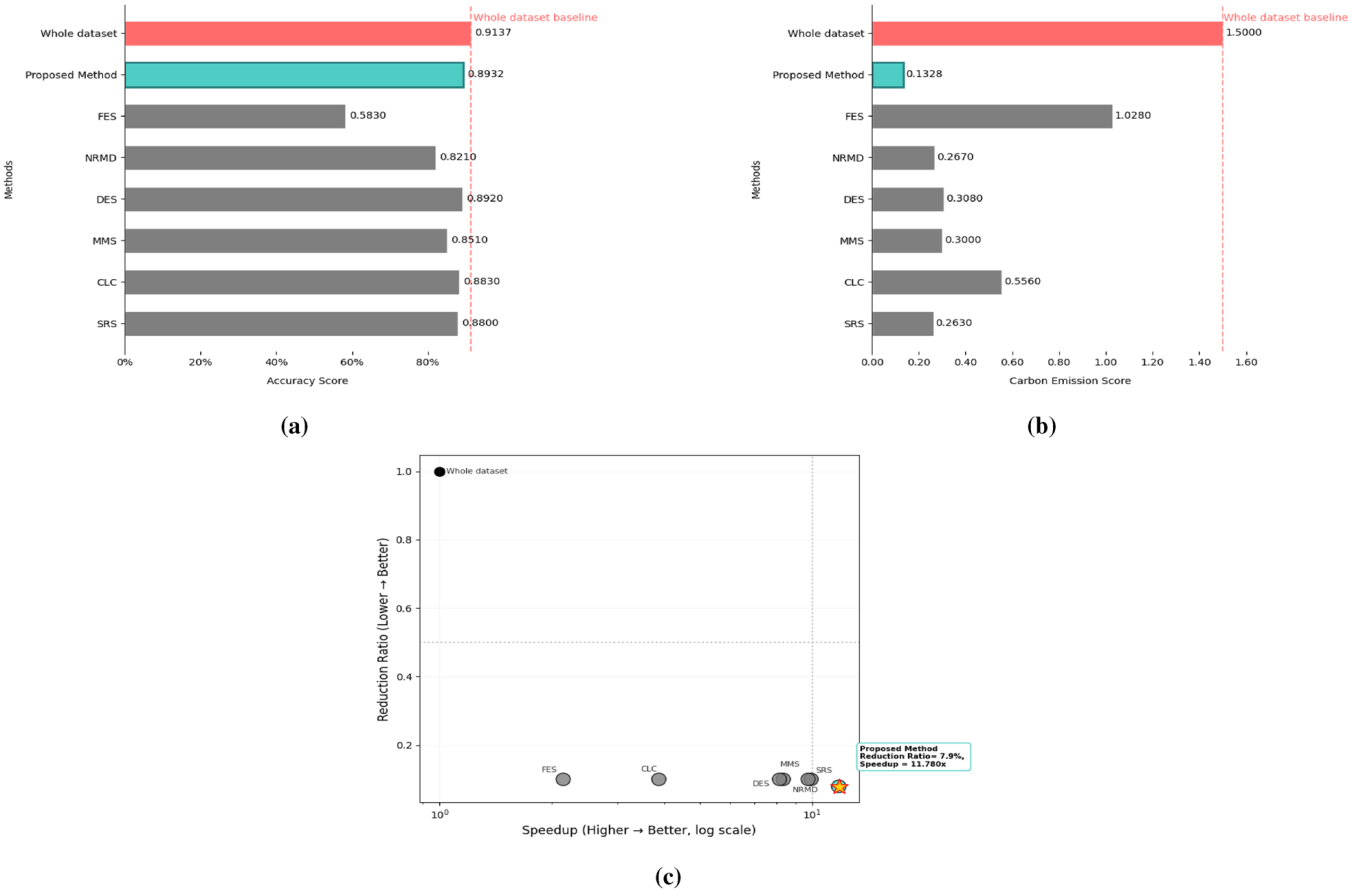
Visualization of collision dataset experimental results: (**a**) accuracy score, (**b**) Carbon emission score and (**c**) Speedup versus accuracy score.

We applied the KM-DBSCAN algorithm directly to the original high-dimensional Collision dataset without performing dimensionality reduction to extract border points and subsequently train the model classifiers using the reduced dataset. The results are summarized in Table 8 and visualized in Fig. 12. The proposed method exemplifies the principle of quality over quantity, achieving superior performance with only 7.9% of the training data (6392 samples vs. 80407 in the training dataset). Despite this drastic reduction, it surpasses all methods by getting closer to the original accuracy (0.8932 vs. 0.9137) while delivering a substantial 11.780$\times$ speedup in computational time (72.4598 s vs. 853.58 s). The environmental footprint is also minimized, with carbon emissions reduced to only 0.1328 g compared to 1.500 g for full training. Notably, even approaches utilizing more data (e.g., 10% reduction methods such as SRS and DES ) fail to match our accuracy or efficiency, achieving speedups of only 8–10$\times$ and accuracy below 0.8932. These results confirm that strategic selection of high-quality border points via KM-DBSCAN is more effective than processing larger volumes of data. The KM-DBSCAN hyperparameters for this dataset were configured as follows: $k = 2000$ for the negative class and $k = 6000$ for the positive class. DBSCAN parameters were tuned separately for each class, with $\varepsilon = 0.87,\, \text {MinPts} = 411$ for the negative class and $\varepsilon = 0.80,\, \text {MinPts} = 437$ for the positive class. The speedup factor for the related work in Table 8 has been calculated according to the execution time measures in the references.

**Table 8:** Results of the KM-DBSCAN data reduction compared to other methods from the related work on the collision dataset.

Method	Accuracy	Loss/Gain in Accuracy	Reduction Ratio	Carbon (g)	Speedup
Whole dataset	0.9137	–	–	1.500	–
SRS^15^	0.8800	− 0.0337	0.100	0.263	9.9276
CLC^15^	0.8830	− 0.0307	0.100	0.556	3.8736
MMS^15^	0.8510	− 0.0627	0.100	0.300	8.3564
DES^15^	0.8920	− 0.0217	0.100	0.308	8.1657
NRMD^15^	0.8210	− 0.0927	0.100	0.267	9.7511
FES^15^	0.5830	− 0.3307	0.100	1.028	2.1457
Proposed method	0.8932	− 0.0205	0.079	0.1328	11.780

**Fig. 13:**
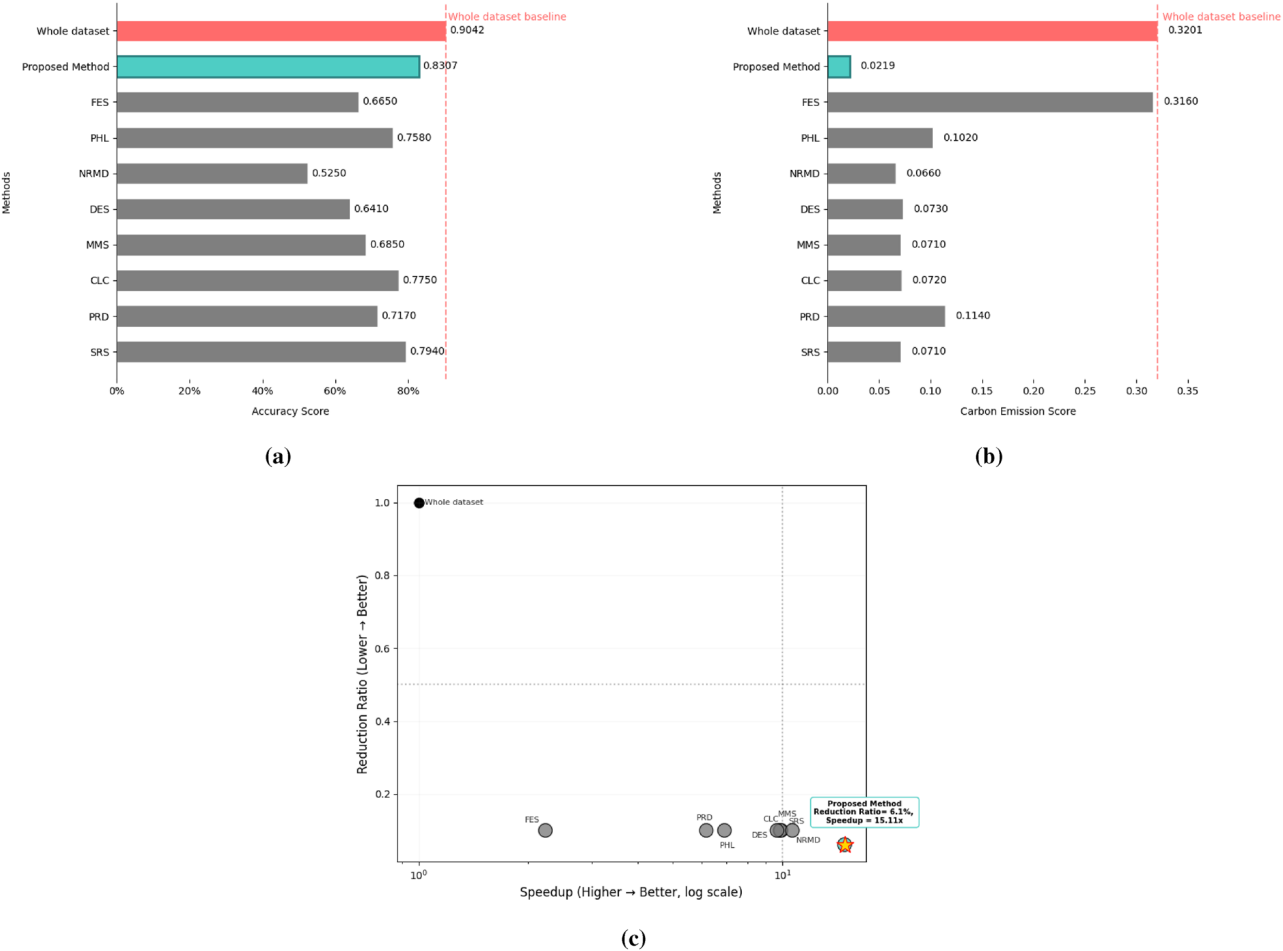
Visualization of dry bean dataset experimental results: (**a**) Accuracy score, (**b**) Carbon emission score and (**c**) Speedup versus accuracy score.

For the Dry Bean dataset, the model was trained for 150 epochs with a batch size of 32. We extended the KM-DBSCAN framework to this multi-class classification benchmark to assess its generalizability beyond collision prediction. The original high-dimensional data were processed without dimensionality reduction to extract discriminative boundary points. The results, presented in Table 9 and Fig. 13, show that the proposed method exemplifies the principle of quality over quantity, achieving superior performance with only 6.1% of the training data (631 samples vs. 10208 in the training dataset). Despite this drastic reduction, it surpasses all methods by getting closer to the original accuracy (0.8307 vs. 0.9042) while delivering a substantial 15.11$\times$ speedup in computational time (10.082 s vs. 152.43 s). The environmental footprint is also minimized, with carbon emissions reduced to only 0.0219 g compared to 0.3201 g for full training. Compared to methods such as NRMD (10.64$\times$ speedup but 0.3792 lower accuracy on the training dataset) and DES (9.65$\times$ speedup with 0.2632 lower accuracy), our approach consistently preserved highly discriminative boundary points in this complex multi-class scenario. The KM-DBSCAN hyperparameters for this dataset were configured as follows: $k = 300$. DBSCAN parameters were tuned separately for each class, with $\varepsilon = 0.16$ and $\text {MinPts} = 20$ for all classes. The speedup factor for the related work in Table 9 has been calculated according to the execution time measures in the references.

**Table 9:** Results of the KM-DBSCAN data reduction compared to other methods from the related work on the dry bean dataset.

Method	Accuracy	Loss/Gain in Accuracy	Reduction Ratio	Carbon (g)	Speedup
Whole dataset	0.9042	–	–	0.3201	–
SRS^15^	0.7940	− 0.1102	0.1000	0.071	9.92015
PRD^15^	0.7170	− 0.1872	0.1000	0.114	6.17391
CLC^15^	0.7750	− 0.1292	0.1000	0.072	9.83671
MMS^15^	0.6850	− 0.2192	0.1000	0.071	9.88071
DES^15^	0.6410	− 0.2632	0.1000	0.073	9.65517
NRMD^15^	0.5250	− 0.3792	0.1000	0.066	10.64239
PHL^15^	0.7580	− 0.1462	0.1000	0.102	6.92682
FES^15^	0.6650	− 0.2392	0.1000	0.316	2.23095
Proposed method	0.8307	− 0.0735	0.061	0.0219	15.1193

### KM-DBSCAN for intelligent data reduction on CNN for early diagnosis of melanoma

**Fig. 14:**
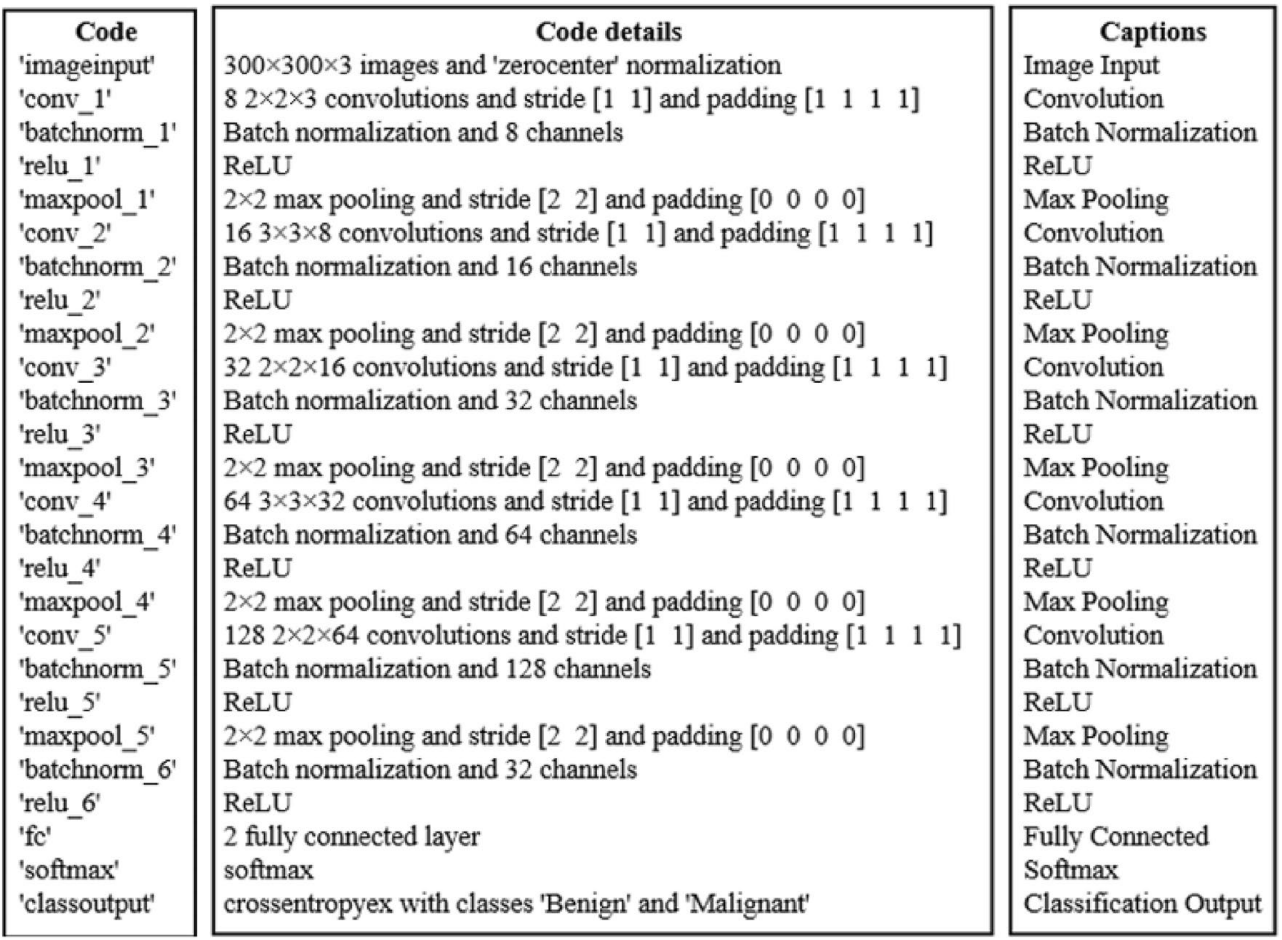
CNN architecture model^8^.

**Fig. 15:**
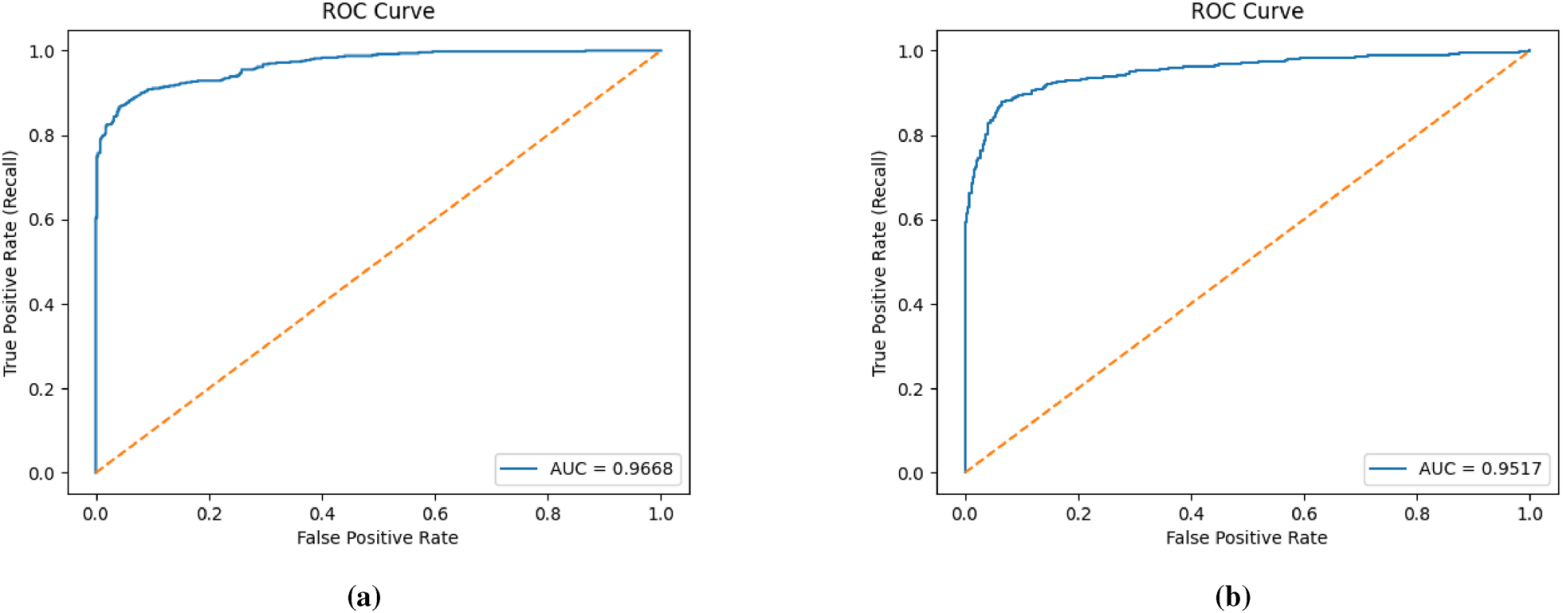
ROC-AUC curves for melanoma classification: (**a**) Full dataset and (**b**) After KM-DBSCAN reduction.

**Fig. 16:**
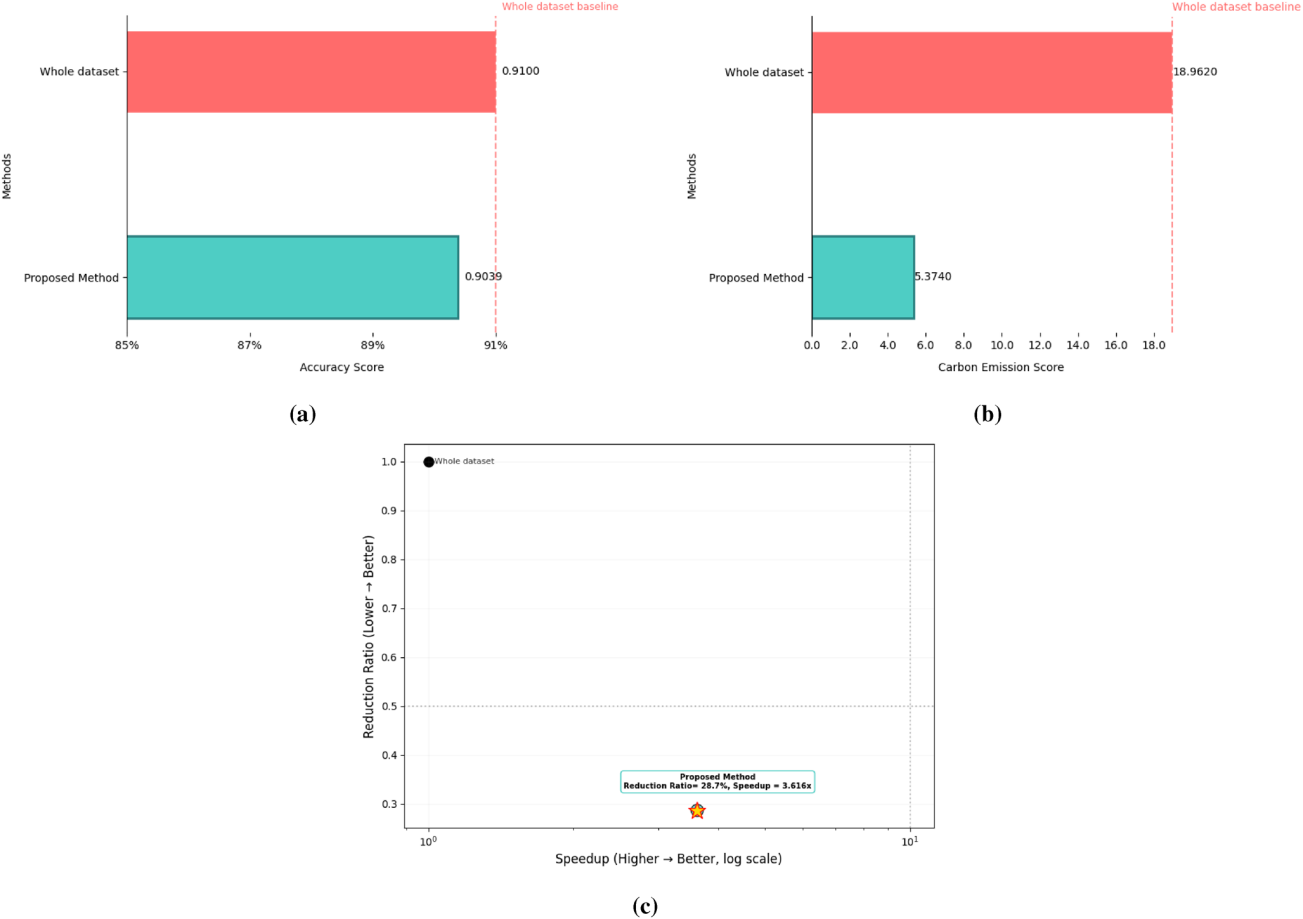
Visualization of melanoma dataset experimental results: (**a**) Accuracy score, (**b**) Carbon emission score and (**c**) Speedup versus accuracy score.

We adopt the CNN architecture proposed by Waheed et al.^8^ for melanoma skin cancer classification, due to its proven performance in handling dermoscopic imagery. Building on this baseline, we introduce an optimized data reduction pipeline that maintains diagnostic accuracy while significantly lowering computational cost. The CNN architecture used in this work processes 300x300x3 dermoscopic images with zero-center normalization. It contains five convolutional blocks, each comprising a convolutional layer, batch normalization, ReLU activation, and max pooling (except for the last block, which omits pooling before the final stage). The convolutional layers progressively increase the number of channels from 8 to 128, capturing hierarchical visual features. A final max pooling layer is followed by a fully connected layer, a softmax activation, and a classification output layer distinguishing between ‘Benign’ and ‘Malignant’ classes (see Fig. 14). For the data reduction, we first flattened each image from its original 3D representation into a 1D vector, then applied UMAP to extract features and reduce dimensionality to 64 dimensions. Next, we applied the KM-DBSCAN algorithm to identify boundary objects, which were subsequently mapped back to the original image space for training a CNN model. As shown in Table 10, our method achieved accuracy comparable to that of the full dataset (0.9039 vs. 0.9100) while using only 28.7% of the training data (2,765 samples vs. 9,605). This demonstrates the framework’s ability to preserve diagnostically critical features in medical imaging analysis. Additionally, the approach yielded a 3.616$\times$ speedup (879.93 s vs. 3,182.14 s) and a 71.65% reduction in carbon emissions (5.374 g vs. 18.962 g). To further evaluate diagnostic robustness, we report the precision, recall, F1-score, and ROC-AUC metrics. The full-data CNN achieved Precision = 0.9278, Recall = 0.8882, and F1-score = 0.9076, compared to Precision = 0.9361, Recall = 0.8638, and F1-score = 0.8985 after our KM-DBSCAN reduction. The results reveal a slight increase in precision (fewer false positives) at the cost of a small decrease in recall (more false negatives), leading to a marginal drop in F1. This trade-off indicates that KM-DBSCAN tends to keep samples that improve class purity (hence better precision) while slightly reducing sensitivity; however, the overall F1 remains comparable, demonstrating that diagnostic utility is largely preserved despite substantial data reduction. Furthermore, ROC-AUC analysis supports this observation: the full-data model achieved an AUC of 0.9668, while the reduced-data model achieved 0.9517, maintaining excellent separability between benign and malignant cases. As illustrated in Fig. 15, the ROC curve remains close to the ideal top-left boundary, indicating strong diagnostic capability even after significant data reduction. These results, visualized in Fig. 16, highlight the clinical applicability of our framework in scenarios where both predictive accuracy and computational efficiency are essential. The KM-DBSCAN hyperparameters for this dataset were configured as follows: $k = 4000$. DBSCAN parameters were tuned separately for each class, with $\varepsilon = 1.0,\, \text {MinPts} = 261$ for the benign class and $\varepsilon = 0.5,\, \text {MinPts} = 76$ for the malignant class.

**Table 10:** Results of the KM-DBSCAN data reduction compared to original model on the melanoma dataset.

Method	Accuracy	Loss/Gain in Accuracy	Reduction Ratio	Execution Time (s)	Carbon (g)	Speedup
Whole dataset	0.9100	–	–	3182.14	18.962	–
Proposed method	0.9039	− 0.0061	0.287	879.93	5.374	3.61635

### Ablation study of KM-DBSCAN components

To demonstrate the efficacy of combining the K-Means and DBSCAN algorithms within the KM-DBSCAN framework, we conducted a detailed Ablation Study, the results of which are presented in Table 11. This study aims to assess the contribution of each component to the overall performance compared to the complete hybrid framework. The results compare the classification accuracy, reduction ratio, and speedup factor across three scenarios: using K-Means for data reduction only, using DBSCAN for data reduction only, and using KM-DBSCAN. The results on the Banana and Collision datasets conclusively show that the hybrid approach is superior; on the Banana dataset, KM-DBSCAN achieved the highest accuracy (0.9107) and the best speedup factor (98.2x), significantly surpassing the results achieved by K-Means alone (31.67x) or DBSCAN alone (8.04x). Furthermore, in relation to the Green AI objective, the proposed framework demonstrated the lowest recorded carbon emissions (0.1328 g CO$_2$) and the highest speedup (11.780x) on the Collision dataset, confirming that the integration of density and centroid based approaches is the optimal strategy for maximizing computational efficiency while maintaining competitive accuracy.

**Table 11:** Ablation study on banana and collision datasets. Each dataset reports accuracy, reduction ratio, computational cost, and a task specific efficiency metric (SVs reduction for banana, carbon emissions for collision).

Method	Accuracy	Reduction ratio	Task specific metric	Time (s)	Speedup
Banana					
Whole dataset	0.9044	–	–	0.0982	–
K-Means only	0.9025	0.107	0.257 (SVs)	0.0031	31.67
DBSCAN only	0.8755	0.255	0.311 (SVs)	0.0122	8.04
KM-DBSCAN	0.9107	0.0372	0.0544 (SVs)	0.0010	98.2
Collision					
Whole dataset	0.9137	–	1.500 (g CO$_2$)	853.58	–
K-Means only	0.8861	0.099	0.1654 (g CO$_2$)	81.888	10.423
DBSCAN only	0.7372	0.110	0.1797 (g CO$_2$)	96.437	8.85
KM-DBSCAN	0.8932	0.079	0.1328 (g CO$_2$)	72.4598	11.780

### Stability and reliability

To assess the stability and reliability of the proposed KM-DBSCAN reduction, each experiment was repeated five times using different random seeds. The average accuracy and the corresponding standard deviation across runs are reported in Table 12. In addition, the table also reports the mean and standard deviation of runtime to evaluate computational stability across repetitions. As shown, the results exhibit high mean accuracy with consistently low variance across all real datasets, indicating strong robustness and insensitivity to random initialization. In particular, datasets such as USPS and Banana demonstrate extremely low fluctuations, confirming that the reduced datasets preserve classification performance in a highly stable manner. Even in more challenging datasets (e.g., DryBean and Melanoma skin cancer), both accuracy and runtime deviations remain small, further validating the reliability and efficiency consistency of the proposed reduction strategy. These results affirm that KM-DBSCAN maintains stable performance and runtime behavior across repeated runs, emphasizing its applicability for real-world scenarios where reproducibility is essential.

**Table 12:** Mean accuracy ± Std over multiple runs for KM-DBSCAN reduced model on the all real dataset.

Dataset	Accuracy Mean ± Std	Runtime (s) Mean ± Std
Banana	$0.9069 \pm 0.0034$	$0.00100 \pm 0.00001$
USPS	$0.9346 \pm 0.0005$	$0.0012 \pm 0.0004$
Adult9a	$0.8263 \pm 0.0158$	$0.0080 \pm 0.0021$
Collision	$0.8652 \pm 0.0058$	$63.38 \pm 0.89$
DryBean	$0.8204 \pm 0.0149$	$8.80 \pm 0.24$
Melanoma Skin Cancer	$0.8784 \pm 0.0100$	$798.64 \pm 20.94$

## Discussion

The experimental findings across six benchmark datasets demonstrate that KM-DBSCAN provides a significant improvement in data reduction efficiency while preserving model performance, thereby reinforcing the central hypothesis of this work: data quality can outweigh data quantity in machine learning training pipelines. The results consistently show that retaining only informative border points leads to substantial computational savings with negligible accuracy degradation, and in several cases even accuracy improvements.

Compared with prior instance-selection approaches such as CBCH, BRIX, and SVOX, KM-DBSCAN delivers higher speedups and lower reduction ratios while maintaining competitive accuracy, particularly on high-dimensional and imbalanced datasets. For example, on the Adult9a dataset, the proposed method achieves a 6907$\times$ speedup while retaining just 2.9% of the data, outperforming BRIX, which achieved 790$\times$ speedup at a 5% dataset size. This suggests that border-focused reduction via density-guided centroid pre-selection provides a more discriminative and compact training subset compared to methods relying solely on geometric heuristics or support-vector neighborhoods.

Results from deep learning experiments further highlight KM-DBSCAN’s suitability for computationally intensive tasks. On the melanoma detection task, a 71.65% reduction in carbon emissions and 3.61$\times$ speedup were achieved while preserving diagnostic accuracy above 90% . This aligns with recent advances emphasizing sustainability in AI pipelines and extends earlier works on Green AI by demonstrating that dataset-level reduction can complement model-level efficiency methods.

Importantly, KM-DBSCAN addresses a well-known limitation in DBSCAN hyperparameter sensitivity by operating on centroid-level representations, leading to stable performance even in overlapping clusters . This behavior contrasts with traditional DBSCAN, where small $\varepsilon$ changes may cause large cluster instability. Furthermore, unlike earlier hybrid methods, the proposed approach offers explicit control over reduction levels, making it practical in resource-constrained deployments such as embedded systems and healthcare imaging.

The design of the proposed KM-DBSCAN framework is grounded in well-established theoretical principles from both centroid-based and density-based clustering, as well as decision boundary analysis in supervised learning. By first compressing the data distribution using K-Means centroids, the method reduces redundancy while preserving the global structure of the data manifold. Applying DBSCAN at the centroid level enables more stable density estimation, as local neighborhood relationships become less sensitive to noise. From a decision-theoretic perspective, retaining only border points aligns with the notion that classification boundaries are primarily determined by samples located near class interfaces, while core points often contribute limited additional discriminative information. This principle is consistent with margin-based learning (e.g., support vectors in SVMs) and is empirically validated in this work across both machine and deep learning models, including CNNs for melanoma classification. Parameter selection in KM-DBSCAN is simplified compared to standard DBSCAN. The centroid-level representation smooth’s the density landscape, making the method less sensitive to variations in the neighborhood radius $\varepsilon$ and *MinPts*. This behavior is confirmed by the sensitivity analysis reported in Tables 3 and 4, where KM-DBSCAN consistently demonstrates greater robustness across a wide range of parameter settings. The framework is also designed to handle challenging boundary cases, such as overlapping classes and heterogeneous density regions. By focusing on centroid-level border structures, KM-DBSCAN avoids common failure modes of DBSCAN in such scenarios, as illustrated in Figs. 4 and 5. In terms of scalability, KM-DBSCAN significantly reduces computational complexity from $\mathscr {O}(n^2)$ in classical DBSCAN to $\mathscr {O}(nk + k^2)$, where $k \ll n$. This theoretical advantage is corroborated by experiments on large-scale datasets, where substantial runtime reductions are achieved without compromising classification performance, supporting the suitability of the proposed method for Green AI–oriented applications.

Overall, these results indicate that KM-DBSCAN advances the state of the art in instance selection by balancing interpretability, controllability, and computational efficiency, paving the way for broader adoption in real-world scenarios where sustainability and scalability are critical.

## Conclusion

This study introduced KM-DBSCAN, a novel hybrid clustering approach that integrates K-means and DBSCAN to enhance density-based data reduction, aiming to support Green AI initiatives. The method first applies a centroid-level preprocessing step using K-means to generate *k* centroids before running DBSCAN. This reduces the computational complexity from $O(n^2)$ in traditional DBSCAN to $O(k^2)$, improves parameter robustness, and better handles overlapping clusters. By operating on a condensed centroid set, DBSCAN’s sensitivity to parameter selection is reduced, and border detection becomes more effective compared with original DBSCAN. The approach was evaluated across six diverse datasets (Banana, USPS, Adult9a, Collision, DryBean, and Melanoma Skin Cancer) using SVM, MLP, and CNN models. KM-DBSCAN strategically retains the most informative border points while discarding redundant core and outlier instances, resulting in substantial dataset size reduction without sacrificing model accuracy. In high-dimensional settings, applying dimensionality reduction (UMAP/PCA) before clustering and mapping selected instances back to the original space preserved essential structures for learning while lowering computational cost. Experimental results show that KM-DBSCAN delivers significant efficiency gains across different model architectures. For SVM, the method achieved up to 284.3$\times$ faster training while maintaining accuracy above 90% on USPS dataset. In MLP-based large-scale tasks, such as vehicle collision prediction, it provided up to 11.78$\times$ speedup, with carbon emissions reduced from 1.5g to 0.1328g. For CNN, applied to melanoma classification, the dataset size was reduced by 28.7% while preserving diagnostic accuracy above 90%.Additional metrics such as precision, recall, F1-score, and AUC-ROC were also considered to ensure clinical relevance. These results consistently outperformed existing methods (FIFDR, CBCH, BPLSH, SE, SVO, SVOX) in reduction ratio, number of support vectors, and environmental metrics. Notably, original DBSCAN was improved by a factor of 57.6$\times$ on the USPS dataset through centroid-based parameter tuning. The findings highlight that in many learning scenarios, data quality can outweigh data quantity. KM-DBSCAN’s alignment with Green AI principles is demonstrated through reduced energy consumption, lower hardware demands, and minimized environmental impact, while maintaining predictive performance. Its versatility across balanced, imbalanced, tabular, and image-based datasets confirms its scalability for real-world AI applications. Overall, KM-DBSCAN offers a sustainable, high-performance solution that addresses key limitations of traditional instance selection and clustering methods. It provides both a theoretical and practical foundation for reducing computational costs without compromising accuracy, paving the way for industrial adoption of environmentally conscious AI systems. Beyond performance gains, KM-DBSCAN offers practical value in real-world machine learning pipelines where efficiency and sustainability are essential. The method is particularly suitable for resource-constrained environments such as edge-AI systems, medical diagnostics, and large-scale industrial analytics, where reducing training cost and energy consumption without compromising accuracy is critical. By enabling controllable instance-level reduction, KM-DBSCAN can support faster model retraining, scalable deployment, and greener AI systems across diverse operational settings.

## Limitations and future work

While KM-DBSCAN demonstrates strong efficiency and performance gains, a few limitations should be acknowledged. First, although KM-DBSCAN is less sensitive to hyperparameter choices compared to classical DBSCAN, the selection of parameters such as the number of clusters *k* in K-Means and the $\varepsilon$/MinPts parameters in DBSCAN may still require dataset-specific tuning. Automating hyperparameter selection remains an important direction for future research. Second, the effectiveness of instance reduction can vary depending on the underlying data distribution, and highly irregular or strongly overlapping class boundaries may influence reduction quality. Finally, since the approach leverages centroid-based compression and density-derived border selection, there is a potential risk of under-representing minority or rare-class samples in highly imbalanced datasets.

Future work will focus on addressing these limitations and enhancing the robustness, fairness, and generalization capability of the proposed KM-DBSCAN framework. Furthermore, we aim to explore the applicability of this framework in diverse domains beyond medical imaging. For instance, integrating our density-based reduction with visual attention models Ishtiaq et al.^33^ could further optimize image processing tasks while maintaining their accuracy. Additionally, the efficiency of KM-DBSCAN in identifying critical data samples could be adapted for large-scale data analytics in cybersecurity and IoT systems Khan et al.^34^, promoting a more sustainable and Green approach across various complex and large-scale real-world datasets.

## Data Availability

The datasets used and/or analysed during the current study are as follows: 1. Banana, available at https://sci2s.ugr.es/keel/dataset.php?cod=182. 2. USPS, available at https://www.kaggle.com/datasets/bistaumanga/usps-dataset. 3. Adult9a, available at https://www.csie.ntu.edu.tw/ cjlin/libsvmtools/datasets/binary.html. 4. Collision, available at https://github.com/Cimagroup/Experiments-SurveyGreenAI. 5. DryBean, available at https://github.com/Cimagroup/Experiments-SurveyGreenAI. 6. Melanoma Skin Cancer, available at https://www.kaggle.com/datasets/hasnainjaved/melanoma-skin-cancer-dataset-of-10000-images. All datasets are publicly available. Further details are available from the corresponding author on reasonable request.

## References

[1] Schwartz, R., Dodge, J., Smith, N. & Etzioni, O. Green AI. *Commun. ACM* **63**, 54–63 (2020).

[2] García, S., Luengo, J. & Herrera, F. Data reduction. In García, S., Luengo, J. & Herrera, F. (eds.) Data Preprocessing in Data Mining, Intelligent Systems Reference Library, 147–162 (Springer International Publishing, Cham, 2015).

[3] Jia, W., Sun, M., Lian, J. & Hou, S. Feature dimensionality reduction: A review. *Complex Intell. Syst.* **8**, 2663–2693. 10.1007/s40747-021-00637-x (2022).

[4] Farouk, M., Sutherland, A. & Shoukry, A. Nonlinearity reduction of manifolds using gaussian blur for handshape recognition based on multi-dimensional grids. In *ICPRAM 2013* (2013).

[5] Farouk, M. Principal component pyramids using image blurring for nonlinearity reduction in hand shape recognition. Ph.D. thesis, Dublin City University (2015).

[6] McInnes, L., Healy, J. & Melville, J. UMAP: Uniform manifold approximation and projection for dimension reduction. arXiv preprint arXiv:1802.03426 (2018).

[7] Xu, X., Liang, T., Zhu, J., Zheng, D. & Sun, T. Review of classical dimensionality reduction and sample selection methods for large-scale data processing. *Neurocomputing* **328**, 5–15. 10.1016/j.neucom.2018.02.100 (2019).

[8] Waheed, S. et al. Melanoma skin cancer classification based on CNN deep learning algorithms. *Malaysian J. Fundam. Appl. Sci.* **19**, 299–305 (2023).

[9] Shen, X. et al. Large-scale support vector machine classification with redundant data reduction. *Neurocomputing* **172**, 189–197. 10.1016/j.neucom.2014.10.102 (2016).

[10] Birzhandi, P. & Youn, H. CBCH (clustering-based convex hull) for reducing training time of support vector machine. *J. Supercomput.* **75**, 5261–5279. 10.1007/s11227-019-02795-9 (2019).

[11] Aslani, M. & Seipel, S. Efficient and decision boundary aware instance selection for support vector machines. *Inf. Sci.* **577**, 579–598. 10.1016/j.ins.2021.07.015 (2021).

[12] Liu, C., Wang, W., Wang, M., Lv, F. & Konan, M. An efficient instance selection algorithm to reconstruct training set for support vector machine. *Knowl.-Based Syst.* **116**, 58–73. 10.1016/j.knosys.2016.10.031 (2017).

[13] Ghaffari, H. Speeding up the testing and training time for the support vector machines with minimal effect on the performance. *J. Supercomput.* **77**, 11390–11409. 10.1007/s11227-021-03729-0 (2021).

[14] Shalaby, M., Farouk, M. & Khater, H. Data reduction for SVM training using density-based border identification. *PLoS ONE* **19**, e0300641 (2024). 38568906 10.1371/journal.pone.0300641 PMC10990207

[15] Perera-Lago, J., Toscano-Duran, V., Paluzo-Hidalgo, E. et al. An in-depth analysis of data reduction methods for sustainable deep learning. *Open Res. Europe*. **4** (2024). 10.12688/openreseurope.17554.2 PMC11413558 39309190

[16] Cherrat, E. M., Alaoui, R. & Bouzahir, H. Improving of fingerprint segmentation images based on k-means and DBSCAN clustering. *Int. J. Electr. Comput. Eng.* **9**, 2425–2432, 10.11591/ijece.v9i4.pp2425-2432 (2019).

[17] Chen, S., Liu, X., Ma, J., Zhao, S. & Hou, X. Parameter selection algorithm of DBSCAN based on k-means two classification algorithm. In *7th International Symposium on Test Automation and Instrumentation (ISTAI 2018) (The Journal of Engineering, Guilin University of Electronic Technology, Guilin, People’s Republic of China, 2018)*. Also affiliated with Jiangsu University, Zhenjiang, People’s Republic of China.

[18] Gholizadeh, N., Saadatfar, H. & Hanafi, N. K-DBSCAN: An improved DBSCAN algorithm for big data. *J. Supercomput.* **77**, 6214–6235. 10.1007/s11227-020-03524-3 (2021).

[19] Naz, A. et al. Short-term electric load and price forecasting using enhanced extreme learning machine optimization in smart grids. *Energies* **12**, 866 (2019).

[20] Haseeb, K., Almustafa, K. M., Jan, Z., Saba, T. & Tariq, U. Secure and energy-aware heuristic routing protocol for wireless sensor network. *IEEE Access* **8**, 163962–163974. 10.1109/ACCESS.2020.3022285 (2020).

[21] Saba, T. Computer vision for microscopic skin cancer diagnosis using handcrafted and non-handcrafted features. *Microsc. Res. Tech.* **84**, 1272–1283 (2021). 33399251 10.1002/jemt.23686

[22] Hastie, T., Tibshirani, R. & Friedman, J. The Elements of Statistical Learning. 2 edn (Springer, New York, NY, 2009).

[23] Pedregosa, F. et al. Scikit-learn: Machine learning in python. *J. Mach. Learn. Res.* **12**, 2825–2830 (2011).

[24] Contributors, C. Codecarbon: A python library for carbon emission quantification. https://codecarbon.io.

[25] Hull, J. A database for handwritten text recognition research. *IEEE Trans. Pattern Anal. Mach. Intell.* **16**, 550–554. 10.1109/34.291440 (1994).

[26] Platt, J. Fast training of support vector machines using sequential minimal optimization. In *Advances in Kernel Methods—Support Vector Learning*, 185–208 (MIT Press, 1998).

[27] Tan, P., Steinbach, M. & Kumar, V. *Introduction to Data Mining*. 1 edn. (Addison-Wesley Longman Publishing Co., Inc, USA, 2005).

[28] Mongelli, M. et al. Performance validation of vehicle platooning through intelligible analytics. *IET Cyber-Phys. Syst. Theory Appl.* **4**, 120–127 (2019).

[29] Dry Bean Dataset. UCI machine learning repository. 10.24432/C50S4B (2020).

[30] Koklu, M. & Ozkan, I. Multiclass classification of dry beans using computer vision and machine learning techniques. *Comput. Electron. Agric.* **174**, 105507 (2020).

[31] Javed, H. Melanoma skin cancer dataset of 10000 images. Kaggle (2020). Available at https://www.kaggle.com/datasets/hasnainjaved/melanoma-skin-cancer-dataset-of-10000-images.

[32] Kingma, D. & Ba, J. Adam: A method for stochastic optimization. arXiv preprint arXiv:1412.6980 (2014).

[33] Ishtiaq, U., Baig, A. K. & Ishtiaque, Z. Modeling visual attention for enhanced image and video processing applications. *Int. J. Theoretical Appl. Comput. Intell.* **2025**, 210–226, 10.65278/IJTACI.2025.11 (2025).

[34] Khan, A. Y. et al. Malicious insider attack detection in IoTs using data analytics. *IEEE Access* **8**, 11743–11753. 10.1109/ACCESS.2019.2959047 (2019).

